# Contrastive learning for enhancing feature extraction in anticancer peptides

**DOI:** 10.1093/bib/bbae220

**Published:** 2024-05-09

**Authors:** Byungjo Lee, Dongkwan Shin

**Affiliations:** Research Institute, National Cancer Center, 323, Ilsan-ro, Ilsandong-gu, Goyang, 10408, Republic of Korea; Research Institute, National Cancer Center, 323, Ilsan-ro, Ilsandong-gu, Goyang, 10408, Republic of Korea; Department of Cancer Biomedical Science, National Cancer Center Graduate School of Cancer Science and Policy, 323, Ilsan-ro, Ilsandong-gu, Goyang, 10408, Republic of Korea

**Keywords:** anticancer peptide, deep learning, contrastive learning, therapeutics screening

## Abstract

Cancer, recognized as a primary cause of death worldwide, has profound health implications and incurs a substantial social burden. Numerous efforts have been made to develop cancer treatments, among which anticancer peptides (ACPs) are garnering recognition for their potential applications. While ACP screening is time-consuming and costly, *in silico* prediction tools provide a way to overcome these challenges. Herein, we present a deep learning model designed to screen ACPs using peptide sequences only. A contrastive learning technique was applied to enhance model performance, yielding better results than a model trained solely on binary classification loss. Furthermore, two independent encoders were employed as a replacement for data augmentation, a technique commonly used in contrastive learning. Our model achieved superior performance on five of six benchmark datasets against previous state-of-the-art models. As prediction tools advance, the potential in peptide-based cancer therapeutics increases, promising a brighter future for oncology research and patient care.

## INTRODUCTION

Cancer, a prominent cause of death worldwide, has severe health impacts and places a substantial burden on society [1]. In 2020, around 19.3 million new cancer cases and 10 million cancer-related fatalities emerged globally [2]. Despite global efforts, cancer burden or mortality increases due to evolving risk factors linked to economic and social development [3, 4]. Therefore, efforts to build a suitable healthcare system for early detection and effective treatment are essential for reducing the societal costs associated with cancer.

Due to advancements in peptide synthesis techniques, the development of new peptide-based therapeutics is experiencing a significant boost [5]. Peptide therapeutics can serve as a potent alternative to small molecules due to their distinctive properties, such as high selectivity, flexible backbones and enhanced permeability [6]. By leveraging these beneficial properties, anticancer peptides (ACPs) have been developed as treatments for various cancers and have successfully made their mark in the market [7, 8]. However, developing peptide drugs remains time-consuming and a labor- and resource-intensive process. In response to these challenges, an *in silico*-based approach could accelerate the discovery of promising ACPs [9].

Researchers have recently developed various *in silico* prediction tools to facilitate the discovery of therapeutic peptide [10–13]. Obtaining a suitable dataset for model training necessitates biological experiments, and this inherent complexity imposes limitations on peptide dataset availability. As a result, peptide-based prediction models are trained using a limited number of samples, consequently restricting prediction performance. Therefore, numerous approaches have been undertaken to extract prominent features from peptide data through preprocessing or diverse learning strategies [14–16]. Concerning ACPs, various models have utilized traditional machine learning or deep learning models and proposed new model structures or featuring engineering methods during data preprocessing [15, 17–28].

To enhance prediction performance, contrastive learning can be employed to feature representation learning, a technique widely utilized in deep learning, to enhance the efficacy of these approaches [29]. This technique adopts closeness between ‘similar’ samples and increases the distance between ‘dissimilar’ samples in the embedding space [30]. Contrastive learning aids in capturing and extracting valuable underlying feature representations by comparing samples in the batch or dataset. To utilize the contrastive learning, data augmentation techniques establish ‘similar’ and ‘dissimilar’ samples: ‘similar’ samples are paired augmented instances from the same original source, while ‘dissimilar’ samples come from distinct original sources [29]. This technique has notably improved model performance in language, vision and audio domains [31–34]. However, augmenting sequences of peptides or proteins, which is crucial for contrastive learning, remains a challenge, and a method to overcome this challenge is required to apply contrastive learning [35].

In this study, we developed an accurate tool for screening ACPs using only peptide sequences. To enhance model prediction performance, contrastive and cross-entropy losses were implemented simultaneously. Our approach uses two distinct encoders in conjugation with two different tokenizers, enabling contrastive learning. This novel setup allows the identical peptide sequence to be transformed into two unique index representations by the respective tokenizers, enhancing the ability of the model to capture and learn underlying feature representations from the data. The outputs from these encoders were transformed into respective classification probability and projection vectors for classification and contrastive loss, respectively. Through experiments to adjust the ratio between CE and contrastive loss, our approach demonstrated superior performance on five out of six benchmark datasets than the most recent models.

## METHODS

### Benchmark dataset

To benchmark performance against previously developed models, we selected a variety of datasets that had been used across multiple prediction models. Among the benchmark datasets utilized for predicting anticancer peptide prediction, we focused our model training on the six benchmark datasets organized by He *et al.* [15, 24, 27, 36, 37]. Table S1 displays the statistics of the six benchmark datasets. These datasets are pre-divided into sections for model training and independent testing. Consequently, sequence redundancy was assessed within the training dataset, within the test dataset and between the training and test datasets. A sequence redundancy was obtained through a basic local alignment search tool (BLAST) homology search within and between each training and test dataset to determine the reliability of each dataset [38]. In each sequence, a query sequence was identified as redundant if it exhibited an e-value of 1E-5 or lower, when compared to any sequence in the database, excluding the sequence itself. In this context, for redundancy within the training or test dataset, the comparison involves the query sequence and the identical dataset as the database. Conversely, when assessing redundancy between the training and test datasets, the comparison is structured such that one dataset serves as the query while the other functions as the database.

### Sequence representation

Peptides are represented as a series of amino acids. There are 21 unique amino acids, including the unspecified amino acid denoted as ‘X’, represented by a distinct letter. The tokenizer transformed the amino acid sequence into input indices to feed peptide data to the model. There were two tokenizers: one that converted a single amino acid into a single index and another that altered amino acid pairs into an index. The first tokenizer ranges from 0 to 22, which transformed a single amino acid into a single index including sequence start token and padding token. The second tokenizer mapped two amino acids into a single index that ranges from 0 to 442, which also includes the sequence start token and padding token. By two tokenizers, the identical peptide sequence altered into two different indices sequences, which gives a data augmentation effect. The indices transformed by the two tokenizers were prepared to be fed into the respective embedding layers, which convert the discrete indices vector into a more compressed vector.

### Model architecture

For contrastive learning, two independent encoders were developed. Each encoder layer was configured to extract a feature representation vector from the output of the embedding layer, where respective tokenizers transformed one or two amino acid–length tokens into corresponding indices. The vector encoded by each tokenizer was input into embedding 1 when a single amino acid–length token was used and into embedding 2 when two tokens were used. The embedded vectors were fed into the respective encoder layers 1 and 2, and the outputs were used for classification and feature projection. The linear head determined the classification result, while the projector layer carried out the feature projection.

Convolutional neural networks (CNNs) have demonstrated considerable success in analyzing spatial data, while long short-term memory (LSTM) networks are specifically tailored to process sequential data, efficiently capturing information from both past and current states within a sequence [39]. Furthermore, the transformer–encoder architecture is renowned for its ability to effectively capture long-term dependencies in natural language data with its self-attention mechanism [40]. The three architectures each have their positive aspects and have already been used in previous research for predicting functional peptides with performance improvements [13, 16, 41–43]. Therefore, these three architectures were implemented for the backbone architecture to evaluate model performance based on the encoder structure. The feature vector by the encoder was fed into a linear head and projector for CE and contrastive loss calculations. Detailed model information is shown in Table S4.

### Model training

For improving classification performance, the cross-entropy loss was calculated as follows:


$${L}_{CE}=-\left(1-\beta \right)\sum_i{y}_i\log{\hat{y}}_{i,1}-\beta \sum_i{y}_i\log{\hat{y}}_{i,2}$$

where $i$ is the index of a selected sample, ${y}_{i,1}$ is the true class label and ${\hat{y}}_{i,1}$ and ${\hat{y}}_{i,2}$ represent the predicted results of linear head 1 and linear head 2 obtained using different tokenizers. The reflection ratio of CE loss from each linear head can be modulated by adjusting the $\beta$ coefficient. When $\beta$ is 0, only the prediction result from linear head 1 is used for calculating CE loss. When $\beta$ is 0.5, linear heads 1 and 2 contribute equally to the CE loss calculation. Lastly, a $\beta$ value of 1 only considers linear head 2.

For effective latent representation extraction, contrastive loss was derived as follows:


$${L}_{CL}=-\sum_i\log \frac{\exp \left({\boldsymbol{z}}_{i,1}\cdot{\boldsymbol{z}}_{i,2}/\tau \right)}{\sum_{i\ne j}\exp \left({\boldsymbol{z}}_{i,1}\cdot{\boldsymbol{z}}_{j,2}/\tau \right)}$$

where $\tau$ is a temperature as well as ${\boldsymbol{z}}_{i,1}$ and ${\boldsymbol{z}}_{i,2}$ are the respective linear head 1 and head 2 outputs. Contrastive loss is computed based on the projection vectors from linear heads 1 and 2, which receive different tokenizer inputs for the model instead of augmented samples. The contrastive loss was not used for pretraining. Instead, cross-entropy and contrastive losses were utilized for model training simultaneously. The final loss was given by:


$${L}_{final}=\left(1-\alpha \right){L}_{CE}+\alpha{L}_{CL}$$

where the $\alpha$ coefficient controls the ratio between cross-entropy loss and contrastive loss in determining the final loss.

Regarding the dataset selection for model performance validation, an independent test set was already predefined in the benchmark dataset. For the validation set during model training, we allocated 10% of the training dataset, with the remaining 90% being utilized for model training. The train–validation split is executed at each training procedure, with the splitting process governed by a specified random seed input. Consequently, the data split is consistently replicated with an identical input random seed, ensuring reproducibility and consistency in the training process.

The model training was conducted using a grid search approach to optimize several parameters of random seed, alpha, beta and temperature. For the learning rate, 1e-3 was chosen as it showed stable training in our training cases. Detailed training information is shown in Table S5. The model training process was implemented using PyTorch [44]. Experiments were executed on a workstation with a central processing unit of AMD Ryzen Threadripper PRO 5955WX, graphic processing unit of GeForce RTX 4090 and 256GB of random access memory.

### Evaluation metrics

Model performance was evaluated based on accuracy, sensitivity, specificity, Matthews correlation coefficient (MCC), receiver operating characteristic (ROC) and the area under the ROC curve (AUC). The definitions are as follows:


$$\text{Accuracy}=\frac{TN+ TP}{TN+ TP+ FN+ FP}$$

$$\text{Sensitivity}=\frac{TP}{TP+ FN}$$

$$\text{Specificity}=\frac{TN}{TN+ FP}$$

$$\text{MCC}=\frac{TP\times TN- FP\times FN}{\sqrt{\left( TP+ FP\right)\left( TP+ FN\right)\left( TN+ FP\right)\left( TN+ FN\right)}}$$

where TP is true positive, TN is true negative, FP is false positive and FN is false negative.

### Model interpretation

The explainable artificial intelligence technique was applied to identify amino acid residues primarily used in the model for predicting ACP. Among techniques, the integrated gradient method (an attribution-based method) was applied using the Captum library [45, 46]. Within the ACP-Mixed-80 test dataset, positive class data were provided and used to compute the attribution score at the embedding layer. These attribution scores were then summarized by calculating the average value for each amino acid residue.

## RESULTS

### Benchmark dataset analysis

For effective training and evaluation of the classification model, it is crucial to consider the composition of samples in the dataset. Numerous models for classifying ACPs and various benchmark datasets have been developed. To assess the suitability of the respective datasets for model training and evaluation, sequence redundancy was reviewed with the BLAST, which identifies sequence similarity between biological sequences [38]. BLAST was conducted within the train (train-to-train), test (test-to-test) and between the train and test datasets (train-to-test and vice versa). In the BLAST results, an e-value below 1E-5 was considered a homologous sequence exhibiting high similarity between sequences. These homologous sequence ratios were verified among the abovementioned comparison groups (Table 1).

**Table 1:** Proportion of significant homologous peptide sequences among datasets from BLAST search results

Dataset	Train-to-train	Train-to-test	Test-to-train	Test-to-test
ACP-Mixed-80	**0.1983**	**0.1116**	**0.1803**	**0.0656**
ACP2.0 main	0.3120	0.2351	0.4012	0.2384
ACP2.0 alternative	0.3492	0.2448	0.4124	0.2577
LEE + Independent	0.2275	0.2299	0.4733	0.1400
ACP500 + ACP2710	0.2560	0.1560	0.2561	0.1707
ACP500 + ACP164	0.2760	0.1640	0.2683	0.2073

The numbers in boldface indicate the lowest proportion of significant homologous peptide sequences.

For model training (train-to-train), the ACP2.0 alternative displayed the highest redundancy, while ACP-Mixed-80 exhibited the lowest within training sequences. Regarding model evaluation, more than 20% of test sequences presented significant homology to the training dataset (test-to-train) in each dataset, excluding ACP-Mixed-80. In particular, the ACP2.0 main, ACP2.0 alternative and LEE + Independent datasets displayed ratios exceeding 40%. Excluding ACP-Mixed-80, more than 10% of test samples contained significantly similar sequences in identical test datasets (test-to-test). A high ratio of sample overlap within the test dataset or between the test and training datasets could skew the assessment of generalization performance, potentially leading to an overly optimistic evaluation of model performance. Consequently, the results will primarily center on ACP-Mixed-80, as it consistently exhibited the lowest sequence redundancy in all cases.

### Model design for contrastive learning

To improve the prediction performance of ACPs, contrastive learning was conducted, which aids in extracting latent representations from limited peptide sequences. When calculating contrastive loss in image datasets, an input sample is augmented to provide positive and negative sample pairs. The contrastive loss is then computed to ensure that the feature vectors of positive pairs are similar while those of negative pairs are dissimilar. This process extracts additional features from the data. However, unlike image datasets, data augmentation is challenging for amino acid sequence–based datasets due to the difficulty of determining whether an augmented amino acid sequence exhibits anticancer activity.

**Figure 1:**
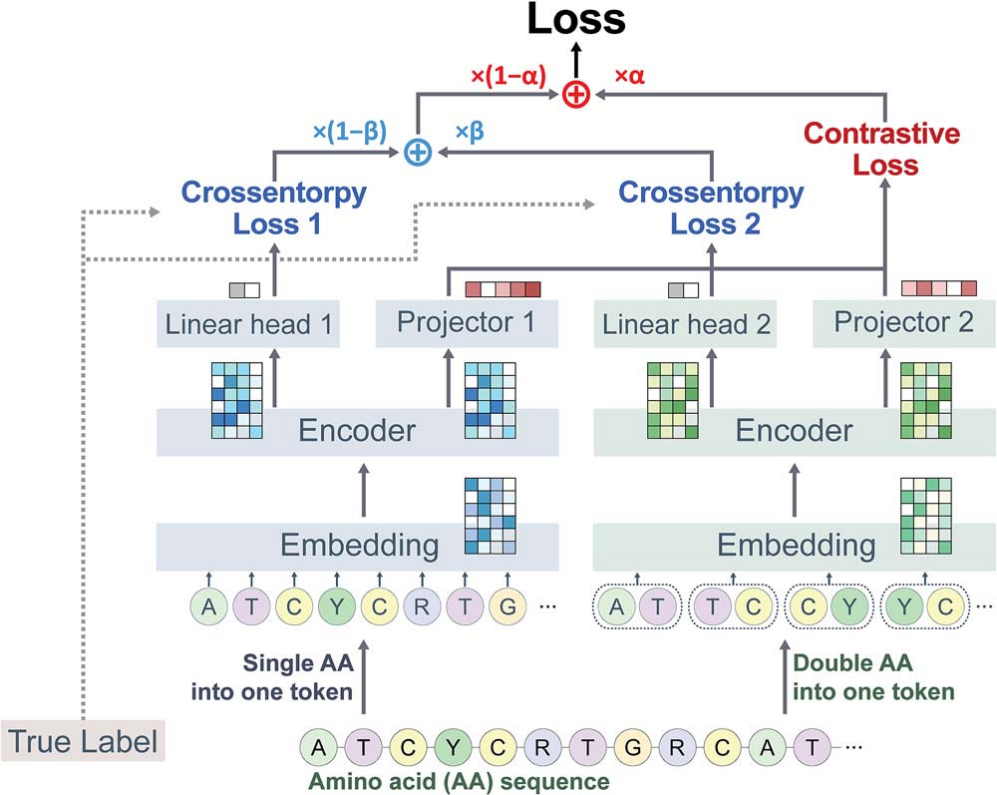
Overview of contrastive learning for classifying anticancer peptides. Each encoder receives an amino acid sequence transformed by its corresponding tokenizer that utilizes single or double amino acid vocabularies. Linear heads 1 and 2 provide classification probabilities, whereas projectors 1 and 2 formulate feature vectors for contrastive learning. The coefficient alpha indicates the extent to which the contrastive loss is reflected; the coefficient beta represents the proportion of CE loss in the classifications predicted by each encoder.

We addressed this issue by developing two distinct models with identical architectures to provide positive and negative dataset pairs, each utilized with a different tokenizer for the equal input peptide sequences (Figure 1). The first tokenizer processes amino acid sequences using a vocabulary of single-length amino acids, while the second tokenizer employs double-length amino acids for tokenization. Each tokenizer converts the identical input sequence into indices, which are then fed into their respective embedding layers of each encoder. Each encoder layer transforms the embedding layer output into a representation vector, and the projection head converts the representation vector into a feature vector. The feature vectors derived from an identical sequence served as positive pairs, while those from distinct sequences formed negative pairs for calculating contrastive loss.

To train the model for classifying ACPs, a combination of both CE and contrastive loss were conducted simultaneously. In our approach, each linear head of both models is employed for classification purposes, leading to the generation of two distinct CE loss values. The coefficient beta is crucial for determining the extent to which the CE loss from either model—using single-length or double-length amino acid tokens—is factored into the training process. Specifically, when the coefficient beta value is 0 or 1, the model training exclusively relies on the CE loss from Model 1 or 2, respectively. Conversely, when the beta is set to 0.5, both CE losses from the two models are employed equally during model training. In addition, a coefficient alpha is introduced to control the balance between CE and contrastive loss components. The alpha coefficient is utilized to adjust CE and contrastive loss weighting during training, allowing for a flexible adjustment of their contributions to the learning process. When the alpha coefficient is set to 0, contrastive learning is excluded from the model training. This method serves as a baseline for comparing model performance when contrastive learning is utilized. To identify the model with optimal performance, we employed a grid search strategy to systematically adjust alpha at values of 0.1, 0.3, 0.5, 0.7 and 0.9 and beta at 0, 0.5 and 1, tuning both parameters simultaneously. This approach allowed us to explore a comprehensive range of configurations to determine the most effective combination for enhancing the model’s predictive accuracy.

### Contrastive learning enhanced prediction performance

Initially, we evaluated the impact of the temperature coefficient on prediction performance, a parameter in the computation of contrastive learning (Figures S1–S6). Except for the ACP500 + ACP2710 and ACP-Mixed-80 datasets, the median value of the MCC metric exhibited an upward trend as the temperature coefficient increased. However, for the ACP500 + ACP2710 and ACP-Mixed-80 datasets, model performance enhanced up to a temperature coefficient of 0.2, beyond which no further improvement was observed.

**Figure 2:**
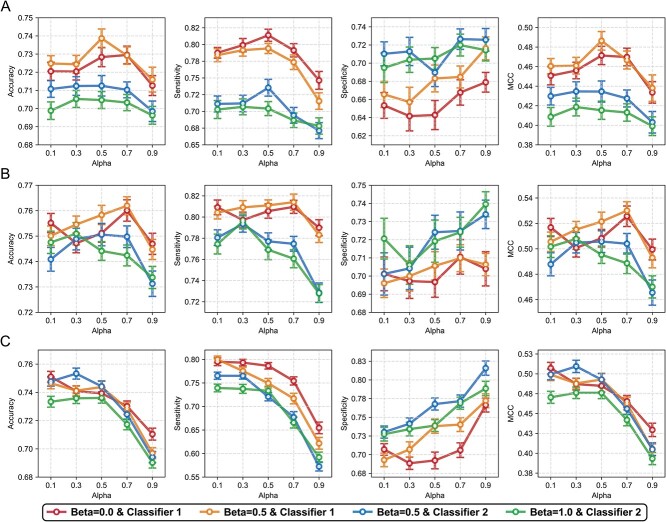
Performance comparison relative to contrastive learning hyperparameters using the ACP-Mixed-80 dataset. Each point denotes the mean of each performance metric trained by contrastive learning relative to the coefficient alpha. The results are plotted on each line corresponding to coefficient beta and classifier type. Error bars represent ± standard error of the mean. The results are displayed by encoder architectures of (**A**) CNN, (**B**) transformer-encoder and (**C**) LSTM.

To investigate the impact of coefficient alpha on model performance, prediction results were averaged for the respective coefficient beta and prediction classifier on the ACP-Mixed-80 dataset (Figure 2). The error bars represent model performance, summarized over training sessions with seven random seeds and five coefficient temperatures. When the encoder was implemented with a CNN, classifier 1 (output of linear head 1 utilizing tokenizer 1 with a single-length amino acid vocabulary) exhibited better accuracy, sensitivity and MCC than classifier 2 (output of linear head 2 utilizing tokenizer 2 with a double-length amino acid vocabulary) (Figure 2A). Conversely, classifier 2 consistently demonstrated higher specificity. Outcomes in the transformer-encoder architecture closely paralleled those of the CNN. In contrast, classifier 2 displayed an inverse correlation between decreased sensitivity and increased specificity as alpha rose (Figure 2B). For LSTM, as the alpha increased, there was a decline in accuracy, MCC and sensitivity, while specificity increased for classifiers 1 and 2 (Figure 2C). On average, classifier 1 outperformed classifier 2 concerning accuracy and MCC when evaluating both positive and negative samples. These differences were reduced in the encoder architecture order CNN, transformer-encoder and LSTM. For other datasets, classifier 1 consistently matched or outperformed classifier 2 in most performance metrics, excluding the LEE + Independent dataset under the transformer-encoder’s architecture (Figures S7–S11). Overall, classifier 1 performed better than classifier 2, irrespective of coefficient alpha.

**Figure 3:**
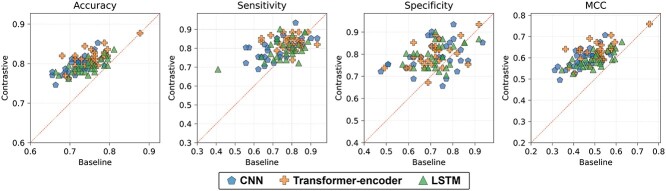
Performance comparison across training methods using the ACP-Mixed-80 dataset with identical architectures and initial weights. Each scatter represents model performance trained by CE (baseline) loss and contrastive learning, displaying performance on the respective *x*- and *y*-axes. The dotted line indicates equivalent performance between baseline and contrastive learning. For the identical model architecture and initial model weight, the area above the line signifies superior performance by contrastive learning, and the area below suggests better performance by the baseline.

In order to investigate performance enhancement via contrastive learning, a model performance comparison was executed between contrastive learning and baseline that excludes contrastive loss on the ACP-Mixed-80 dataset (Figure 3). Each point on the scatterplot depicts model performance when the model was trained exclusively with CE (baseline, represented on the *x*-axis) versus contrastive learning (represented on the *y*-axis), given the identical model architecture and initial model weights. Scatter points predominantly lay above the red line, suggesting that contrastive learning generally improved performance metrics over the baseline. These enhancements are observed across every performance metric of accuracy, sensitivity, specificity and MCC.

Similarly, results from other datasets predominantly exhibited scatters above the line for every metric, excluding sensitivity in the ACP500 + ACP2710 dataset (Figure S12). While maintaining identical model architecture and initial model weight, contrastive learning improved performance against the baseline (Table S2). When working with the ACP-Mixed-80 dataset, almost all models demonstrated improved performance via contrastive learning compared with the baseline concerning accuracy and the MCC. Moreover, over half of the models exhibited enhanced performance in sensitivity and specificity, regardless of encoder architecture. Among other datasets, ACP500 + ACP2710 sensitivity was adversely affected by contrastive learning, with only around 30% of models enhancing performance for every encoder architecture. However, contrastive learning consistently led to improved model performance in other metrics. In all other cases, contrastive learning improved performance in more than half of the models, regardless of the model encoder architecture. Compared to the baseline, contrastive learning tends to improve model performance across all datasets in accuracy and MCC, the primary indicators of overall model performance.

### Performance comparison against state of the art

**Figure 4:**
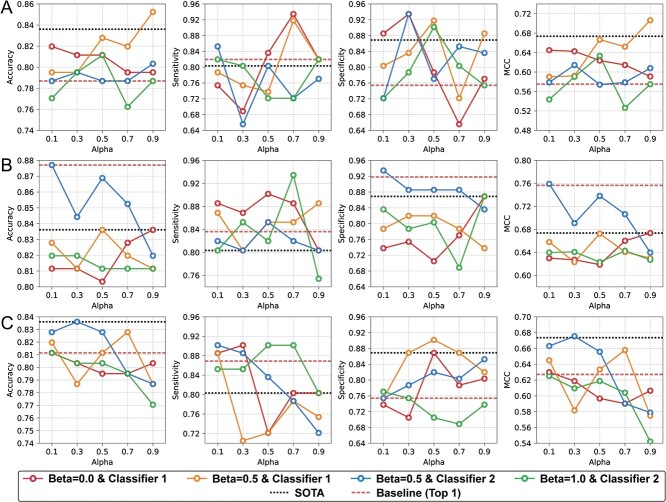
The best model performance relative to each coefficient alpha and beta using the ACP-Mixed-80 dataset. The best model was selected based on the alpha and beta coefficients that achieved the highest MCC metric values. Line plots are presented for each beta coefficient and prediction classifier per coefficient alpha. Dashed lines represent the SOTA and best performances of the baseline. The results are displayed based on the encoder architectures of (**A**) CNN, (**B**) transformer-encoder and (**C**) LSTM.

To assess performance improvements relative to state-of-the-art (SOTA) models, we analyzed model performances according to alpha and beta coefficients against SOTA models [14, 15, 17, 18, 23, 25, 27, 36, 37, 47–52]. The model exhibiting the highest MCC was selected within each encoder architecture, alpha coefficients and beta coefficient, and the performance of these selected models using the ACP-Mixed-80 dataset is exhibited in Figure 4. The model surpassed SOTA in accuracy, specificity and MCC with the CNN encoder when the alpha was set at 0.9, beta at 0.5 and classifier 1 (Figure 4A). When the model was trained using CE loss only, the model outperformed SOTA in sensitivity, while SOTA excelled in other metrics. Concerning the transformer-encoder, the model achieved SOTA in every performance metric when parameters were set with alphas of 0.1, 0.3, 0.5, 0.7 and 0.9; beta at 0.5; and using classifier 2 (Figure 4B). While CE only surpassed SOTA performance, employing contrastive learning yielded even better results in accuracy, MCC and specificity with the parameters alpha as 0.1, beta as 0.5 and utilizing classifier 2. Figures S13–S17 present the results when using other datasets for model training. While our proposed models surpassed SOTA in sensitivity in the AntiCP2.0 main dataset, SOTA achieved better accuracy, specificity and MCC (Figure S13). When assessed with other datasets, contrastive learning consistently improved model metrics in comparison with the SOTA benchmarks (Figures S14–S17).

To select the optimized model, we chose models from both contrastive learning and baseline for each encoder architecture based on their highest MCC metric values on the test dataset (Table 2). In the case of ACP-Mixed-80, the transformer-encoder achieved the top MCC metric value of 0.7591 when trained by contrastive learning. The baseline setting of alpha at 0 closely followed these results with the second-best MCC value of 0.7566. Both configurations outperformed SOTA across all metrics and demonstrated predominant performance relative to other encoder architectures, excluding sensitivity. Although LSTM exhibited the first- and second-highest sensitivity values, its reduced specificity metrics resulted in comparatively diminished accuracy and MCC metric values. For other datasets, the models trained with contrastive learning attained the highest MCC metric values relative to the baseline, surpassing SOTA MCC values, excluding the AntiCP2.0 main dataset. Overall, contrastive learning elevated specificity within identical encoder architecture except for CNN in AntiCP2.0 main, while the trade-off diminished sensitivity. There are detailed comparison results in Table S3.

**Table 2:** Optimized model performance results for respective model architecture, training strategies and pre-SOTA

Dataset	Encoder	Alpha	Beta	AA token length	Accuracy	Sensitivity	Specificity	MCC
ACP-Mixed-80	SOTA [36]	–	–	–	0.8361	0.8033	0.8689	0.6736
	CNN	0	0	1	0.7869	0.8197	0.7541	0.5750
		0.9	0.5	1	0.8525	0.8197	0.8852	0.7064
	Transformer-encoder	0	1	2	**0.8770**	0.8361	0.9180	0.7566
		0.1	0.5	2	**0.8770**	0.8197	**0.9344**	**0.7591**
	LSTM	0	1	2	0.8115	0.8689	0.7541	0.6271
		0.3	0.5	2	0.8361	**0.8852**	0.7869	0.6754
AntiCP 2.0 main	SOTA [50]	–	–	–	**0.8250**	0.7260	**0.9030**	**0.6460**
	CNN	0	0	1	0.7558	0.7326	0.7791	0.5122
		0.1	0.5	1	0.7791	0.8023	0.7558	0.5587
	Transformer-encoder	0	0	1	0.7645	**0.8721**	0.6570	0.5418
		0.1	0.5	2	0.8081	0.8023	0.8140	0.6163
	LSTM	0	1	2	0.7529	0.7209	0.7849	0.5069
		0.1	0.5	2	0.7587	0.6919	0.8256	0.5221
AntiCP 2.0 alter. Dataset	SOTA [51]	–	–	–	**0.9430**	**0.9640**	0.9210	0.8770
	CNN	0	0	1	0.9356	0.9124	0.9588	0.8721
		0.1	0	1	0.9381	0.8969	**0.9794**	**0.8793**
	Transformer-encoder	0	0	1	0.9253	0.9227	0.9278	0.8505
		0.7	0	1	0.9356	0.9227	0.9485	0.8714
	LSTM	0	0	1	0.9201	0.9124	0.9278	0.8403
		0.7	0	1	0.9330	0.9278	0.9381	0.8660
ACP500 + ACP164	SOTA [36]	–	–	–	**0.9146**	0.8902	0.9390	0.8303
	CNN	0	0	1	0.7866	0.7317	0.8415	0.5767
		0.7	0.5	1	0.8354	0.8049	0.8659	0.6720
	Transformer-encoder	0	0	1	0.8780	**0.9146**	0.8415	0.7581
		0.3	0	1	**0.9146**	0.8659	**0.9634**	**0.8332**
	LSTM	0	0	1	0.8110	0.7683	0.8537	0.6242
		0.3	0	1	0.8293	0.8049	0.8537	0.6593
ACP500 + ACP2710	SOTA [36]	–	–	–	0.9358	0.5244	0.9468	0.3271
	CNN	0	0	1	0.9236	0.5244	0.9361	0.2944
		0.9	0.5	1	0.9605	0.4878	0.9753	**0.4110**
	Transformer-encoder	0	0	1	0.8760	**0.6951**	0.8817	0.2884
		0.3	0.5	1	**0.9708**	0.2683	**0.9928**	0.3664
	LSTM	0	0	1	0.8952	0.5732	0.9053	0.2627
		0.3	0	1	0.9185	0.5732	0.9292	0.3070
LEE + Independent	SOTA [36]	–	–	–	0.9667	0.9467	0.9867	0.9341
	CNN	0	1	2	0.9733	0.9600	0.9867	0.9470
		0.1	0.5	1	**0.9767**	0.9533	**1.0000**	**0.9544**
	Transformer-encoder	0	1	2	0.9667	0.9467	0.9867	0.9341
		0.5	0	1	**0.9767**	**0.9667**	0.9867	0.9535
	LSTM	0	0	1	0.9633	0.9533	0.9733	0.9269
		0.1	0.5	1	0.9733	**0.9667**	0.9800	0.9468

The best and second-best performances were bolded and underlined, respectively.

Lastly, we considered the model exhibiting the highest MCC metric values as the optimized model for the datasets. Figure S18 presents the ROC curves of the optimized models for each dataset. These models attained area under the curve values 0.9366, 0.8377, 0.9678, 0.9453, 0.7941 and 0.9822 for ACP-Mixed-80, AntiCP2.0 main, AntiCP2.0 alternative, ACP500 + ACP164, ACP500 + ACP2710 and LEE + Independent datasets, respectively.

### Model interpretation

**Figure 5:**
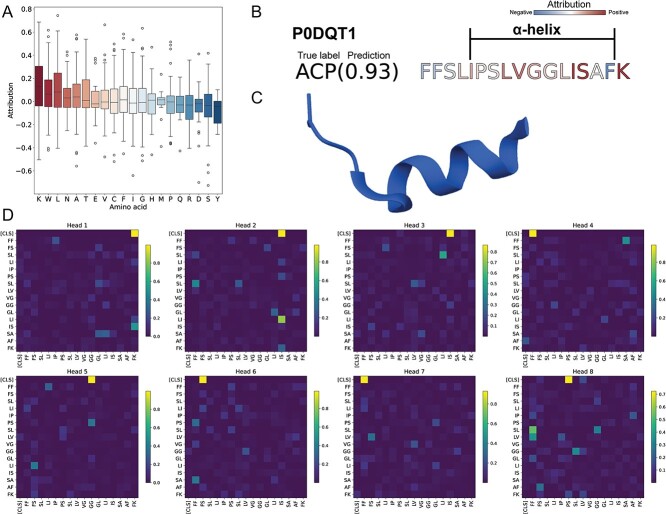
Model interpretability study using the ACP-Mixed-80 test dataset. (**A**) A boxplot that illustrates the attribution scores of individual amino acids for classifying anticancer peptides (ACPs). Amino acids are arranged in descending order based on their attribution mean. (**B**) An example of attribution for stigmurin (Uniprot ID: P0DQT1) is provided. The model predicted the peptide as an ACP with a 0.93 probability, and alpha-helix amino acids predominately displayed high attribution scores. (**C**) The peptide structure of stigmurin obtained from the PDB database (PDB ID: 7KDQ) is depicted. (**D**) Attention scores from various attention heads of the first transformer-encoder layer are presented. Different heads demonstrate distinct preferences for certain amino acid tokens, such as heads 2 and 3 focusing on ‘IS’ and head 5 on ‘GG’.

In order to evaluate the importance of specific amino acid residues in classifying ACPs from input data, we performed a systematic model interpretation via the integrated gradient method. This attribution-based explainable artificial intelligence identifies the predominant amino acids contributing to predictions by calculating the attribution within the embedding layer. The method provided attribution scores that indicated how individual amino acids affected model predictions. We used the ACP-Mixed-80 test dataset to identify the amino acid residues with the most influence on ACP prediction using the optimization model (Figure 5A). Based on the results result, lysine (Lys, K), tryptophan (Trp, W) and leucine (Leu, L) prominently contributed to the accurate prediction of ACP by the optimized model; aspartic acid (Asp, D), serine (Ser, S) and tyrosine (Tyr, Y) exhibited low attribution scores.

Stigmurin (Uniprot ID P0DQT1), known for exhibiting anticancer, antibacterial and antifungal activities, was investigated to determine the contribution of amino acid residues, which were included in the ACP-Mixed-80 test dataset [53, 54]. Stigmurin exhibited an α-helical conformation and was predicted as the ACP with a 0.93 probability due to an overall positive attribution score in the α-helical region (Figure 5B and C). As peptides in the α-helical form can penetrate cancer cells, this characteristic was taken into account by the model when predicting ACPs, with a particular emphasis on the α-helix portion [6]. In addition, we examined which amino acid tokens were primarily considered in the first attention layer of the transformer-encoder, which received output from the embedding layer (Figure 5D). It was found that each head in the model captured distinct self-attention patterns, with a significant focus on amino acid pairs, particularly phenylalanine-lysine (FK), isoleucine-serine (IS), phenylalanine-phenylalanine (FF), glycine-glycine (GG), phenylalanine-serine (FS) and proline-serine (PS), with the [CLS] token. As another example, M-zodatoxic-Lt2a was also studied as it possesses a two-α-helix structure and exhibits anticancer and antibacterial activities [55–57]. The amino acid residues forming the α-helix had a high attribution score in the optimization model, identifying them as ACPs with a 0.96 probability (Figure S19A). Consistent with prior results, each head captured a distinct self-attention pattern (Figure S19B).

## DISCUSSION

This study presented end-to-end deep learning models for classifying ACPs, demonstrating significant performance enhancement with our suggested contrastive learning method. Contrastive learning, a self-supervised representation learning, operates by pairing datasets to closely align features of positive pairs while distancing those of negative pairs, subsequently learning meaningful representations in data [30]. Self-supervised contrastive learning often requires data augmentation; however, due to their properties, a data augmentation process is not readily applicable for peptides. Therefore, two identical models were constructed that exclusively utilized different embedding layers, each processing different indices of the identical peptide sequence through different tokenizers. This dual-encoder configuration allows our model to integrate diverse representations of the peptide sequences, suggesting the effect of data augmentation by providing varied perspectives on the identical sequences. These varied representations are crucial for our contrastive learning framework, where the objective is to learn a feature space that maximally distinguishes between different samples. Instead of traditional data augmentation methods, which were not suitable for our peptide datasets, this innovative approach using dual encoders allows us to exploit the inherent variability of the sequences. This method possibly enhances the generalization ability and robustness, similar to the goals of traditional data augmentation, by providing it with a more complex feature set.

There were six benchmark datasets established for ACP, comprising 606–3210 samples, which is relatively less compared with other deep learning applications (Table S1). Among them, ACP500 + ACP2710 had an imbalance between positive and negative samples in the test data, making the accuracy metric an inadequate measure of model performance. Since at least 25% of samples in the test dataset were significantly similar to the training dataset samples in other benchmark datasets except for ACP-Mixed-80, ACP-Mixed-80 could be presented as the most unbiased performance indicator (Table 1).

For measuring the model performance, accuracy, sensitivity, specificity and MCC were mainly suggested. These metrics were selected because they are commonly measured in previous research, allowing for a consistent basis for comparing model performance. In addition, it is crucial to minimize the false-positive rate among predictions identified as ACPs for subsequent experimental validation. By enhancing the reliability of the identified ACPs, cost losses associated with the experimental process can be reduced. This is achieved by decreasing the likelihood of investing resources in peptides that do not demonstrate anticancer effects during experimental validation, thereby optimizing the efficiency and cost-effectiveness of the research. From this perspective, since the precision metric indicates performance, we also present the performance of the recall metric in Table S3.

To assess the impact of contrastive loss on model training, we evaluated model performance according to the coefficient alpha. In our analysis using the ACP-Mixed-80 dataset, we observed that as the coefficient alpha increased to 0.7 or 0.9, reflecting a higher contrastive loss ratio, there was a decrease in sensitivity and an increase in specificity (Figure 2). This trend was also observed in the transformer encoder and when the LSTM was applied to the ACP500 + ACP2710 dataset. Comparatively, high alpha values decreased sensitivity and specificity metrics in other benchmark datasets (Figures S7–S11). However, in every case, a lower MCC metric value was observed at a 0.9 alpha value compared with other alpha values, indicating that a high contrastive loss ratio negatively impacted model performance. Despite the increase in specificity and accuracy with higher alpha values in the transformer-encoder and LSTM for the ACP500 + ACP2710 dataset, this trend was potentially skewed by the data imbalance issue [58]. Therefore, we used the MCC metric as it offers a more accurate measure when the dataset is imbalanced to evaluate overall model performance more comprehensively. In contrast, we observed that the accuracy metric exhibited a parallel trend in the remaining balance datasets compared to the MCC metric.

To evaluate the effectiveness of contrastive learning for model training, we compared model performance trained through contrastive learning against a baseline from training a model with CE loss only. In addition, the models were trained with seven fixed random seed values for contrastive learning and baseline to ensure consistent model performance due to initial weight settings. This method allowed for a direct performance comparison between contrastive learning and baseline, with learning initiated from identical model weights. In most cases, contrastive learning improved model performance compared with the baseline regardless of encoder architecture and benchmark datasets (Table S2). However, over 65% of models exhibited reduced sensitivity in classifying ACP500 + ACP2710 when contrastive learning was compared with the baseline. Contrastive learning enhanced the accurate identification of negative samples as true negatives while potentially diminishing the efficacy of classifying positive samples as true positives.

To benchmark our model against SOTA models, the best-performing models among respective encoder architectures and training methods were selected based on the highest MCC metric values. Superior performance relative to SOTA models was attained across all benchmark datasets, excluding the ACP2.0 main dataset (Table 2). Furthermore, every case with the highest performance in the MCC metric, which assessed overall performance within each dataset, was consistently associated with contrastive learning. The optimized model that achieved the highest MCC performance outperformed SOTA models across all metrics in ACP-Mixed-80 and LEE + Independent datasets. However, for ACP500 + ACP164, ACP500 + ACP2710 and AntiCP 2.0 alternative datasets, the optimized model displayed reduced sensitivity and increased specificity compared to SOTA models. Additionally, in the comparison between baseline and contrastive learning across all datasets, specificity was consistently maintained or increased across all encoder architectures, whereas sensitivity experienced a decrease in certain instances. This outcome may be attributed to the representation learning process through contrastive learning, which facilitates a deeper understanding of the characteristics inherent in the broader and more diverse negative dataset in the real world.

To dissect the prediction process of the model, we employed explainable artificial intelligence techniques on the embedding layer, enabling us to understand the contribution of each input amino acid. Lysine, tryptophan and leucine emerged as the most substantial contributions to ACP classification with the ACP-Mixed-80 dataset (Figure 5A). Lysine and leucine are recognized for their involvement in cell membrane disruption, while tryptophan is associated with cancer cell metabolism [59–62]. However, this outcome does not imply a direct increase in ACP probability by the optimized model due to the presence of these amino acids. The model considers the sequence context, meaning prediction results are influenced by the surrounding amino acid residues and their interactions. Consequently, the boxplot results for amino acids exhibited significant variance due to the sequential arrangement of amino acids. For specific peptide cases, our attention was focused on the α-helix secondary structure (Figure 5B and Figure S19A). In the ACP-Mixed-80 dataset, more than 80.5% of the ACPs had α-helix structure and 13.9% had β-sheets when the structures were predicted by alphafold [63] (Supplementary Data). This notable occurrence of the α-helix structure is consistent with established research highlighting its importance in ACPs, supporting the significance of α-helix structure in the attribution score of our model.

While the model shows proficiency in cancer anticancer functionality prediction, it encounters difficulties when tasked with pinpointing specific cancer types. This limitation stems from the number of known anticancer peptides for specific types of cancer is insufficient for learning, which is crucial for effective learning. The predicted ACPs might be specificity in its anticancer activity, potentially targeting certain cancer subtypes. Therefore, our model serves as a screening tool for potential ACPs, a crucial step in the early stages of therapeutic development, facilitating the rapid identification of promising peptide drug leads. Specifically, given the abundance of beneficial peptides in animal venoms, our model is capable of accelerating the screening of numerous potential ACPs derived from the venoms [64]. To advance these peptides toward therapeutic applications, the screened candidates must undergo further experimental validation and optimization. Our model can significantly speed up the discovery of promising peptide drug leads for anticancer therapies, enhancing the efficiency of the initial phase of the drug development process.

In the current landscape of peptide research, there is a growing interest in discovering peptides with diverse functional properties, such as anti-cancer, antibacterial, antifungal and anti-inflammatory activities. Subsequently, numerous *in silico*–based methods are being developed to facilitate this endeavor. However, with the inherent nature of the biological field, positive datasets can only be derived from biological experiments, imposing significant constraints on the size of datasets available for model training. The contrastive learning approach developed in this study overcomes these limitations and has demonstrated the potential to produce more precise predictive models than existing methods, as confirmed by our results in ACP prediction. To enhance the application of contrastive learning for ACPs, supervised contrastive learning could be a feasible option. Whereas our current method treats identical samples as similar for contrastive learning, without considering their class, supervised contrastive learning can define similar samples as those belonging to the same class. In addition, with the appropriate data augmentation, rather than our approach, it would enable the use of more diverse sequences for contrastive learning. These methods could facilitate extracting general features specific to each class, potentially improving predictive performance.

**Key Points**

- The paper focused on developing an *in silico*–based tool to predict ACPs, emphasizing the need for effective cancer treatments.
- This study notably applies contrastive learning to peptide prediction models, achieving superior performance in ACP prediction over state-of-the-art methods, as validated by multiple evaluation metrics.
- This paper utilizes explainable artificial intelligence techniques to understand the influence of specific amino acids on the prediction of ACPs, providing insights into the model’s decision-making process.

## Supplementary Material

2_supplementary_bbae220

## CODE AVAILABILITY

The code, implemented in PyTorch, is available at GitHub (http://github.com/bzlee-bio/con_ACP).

## Author Biographies

**Byungjo Lee** is a postdoctoral researcher at Research Institute of National Cancer Center in Republic of Korea. His research interests focus on AI-driven drug target discovery.

**Dongkwan Shin** is a senior researcher at Research Institute of National Cancer Center in Republic of Korea. His research interests focus on cancer systems biology.
