# Supplementary material for: Contrastive learning for enhancing feature extraction in anticancer peptides
Source: Brief Bioinform. 2024 May 9;25(3):bbae220. doi: 10.1093/bib/bbae220 (PMC11082072; doi:10.1093/bib/bbae220)
Supplement: 2_supplementary_bbae220 [file 2_supplementary_bbae220.docx]

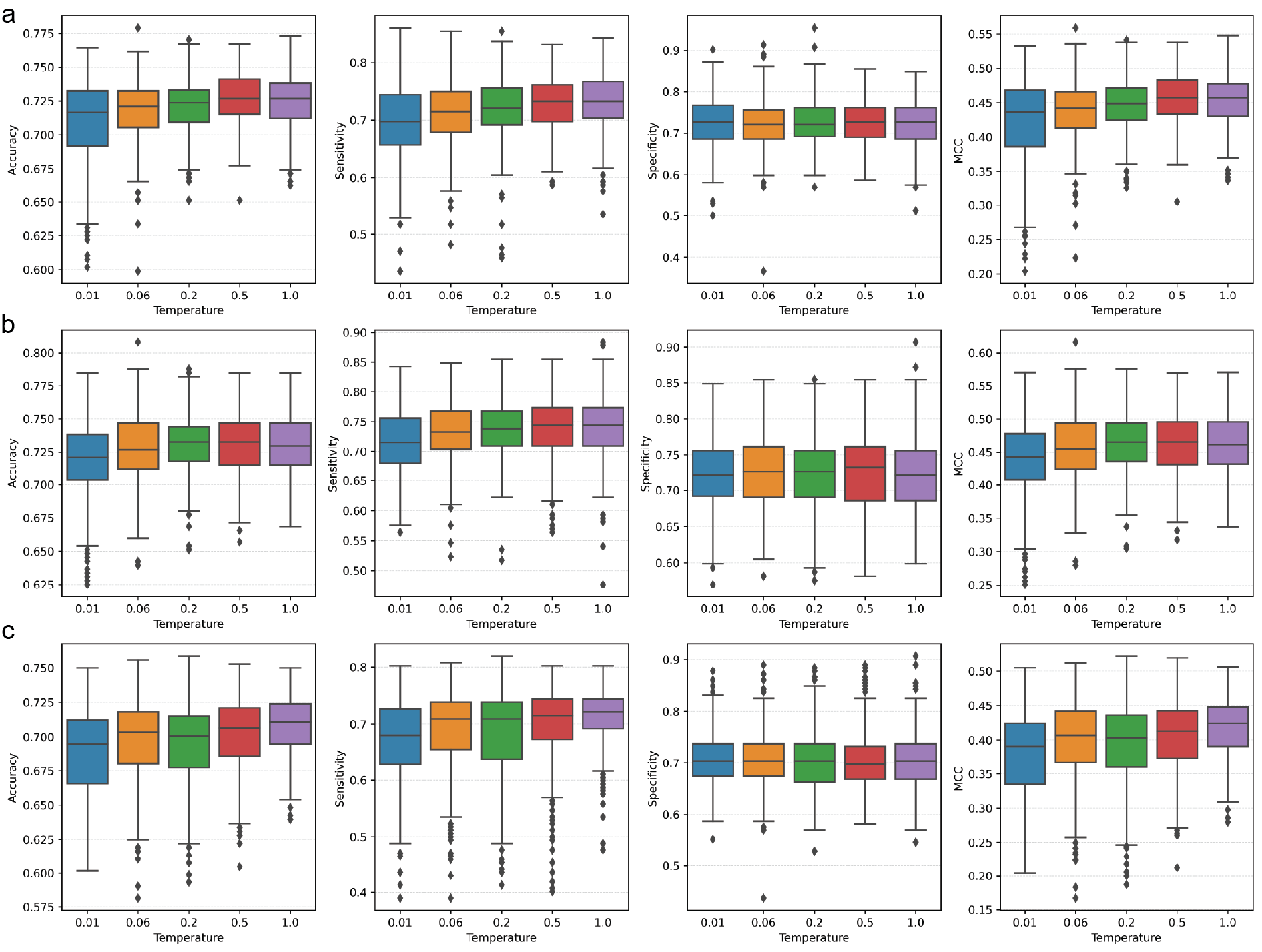


**Figure S1. Performance comparison relative to temperature coefficients using the ACP2.0 main dataset.** Each bar plot denotes the mean of each performance metric trained by contrastive learning relative to the coefficient temperature. Error bars represent ± standard error of the mean. The results are displayed by encoder architectures of **(a)** convolutional neural network (CNN), **(b)** transformer-encoder, and **(c)** long short-term memory (LSTM).


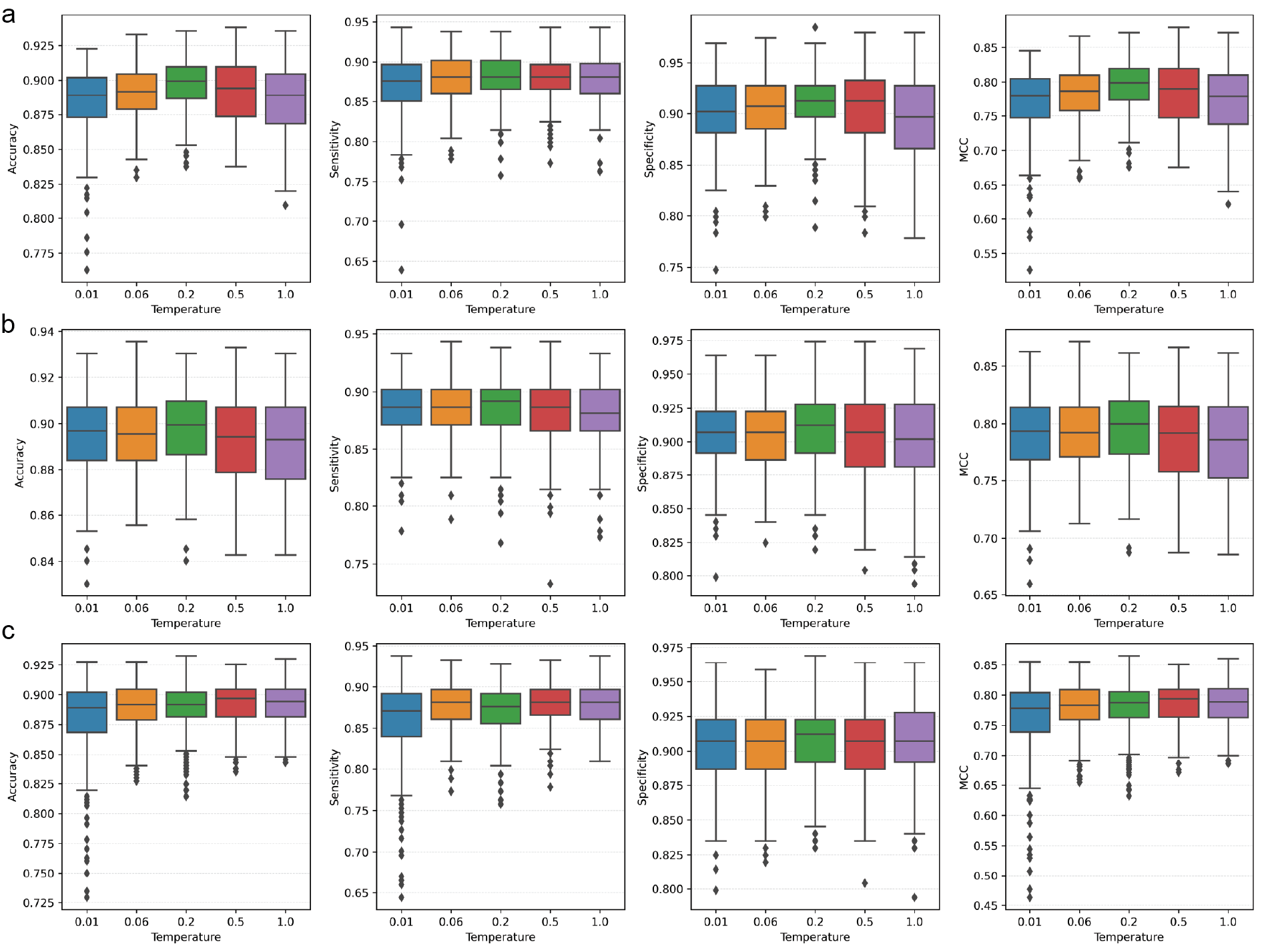


**Figure S2. Performance comparison relative to temperature coefficients using the ACP2.0 alternative dataset.** Each bar plot denotes the mean of each performance metric trained by contrastive learning relative to the coefficient temperature. Error bars represent ± standard error of the mean. The results are displayed by encoder architectures of **(a)** convolutional neural network (CNN), **(b)** transformer-encoder, and **(c)** long short-term memory (LSTM).


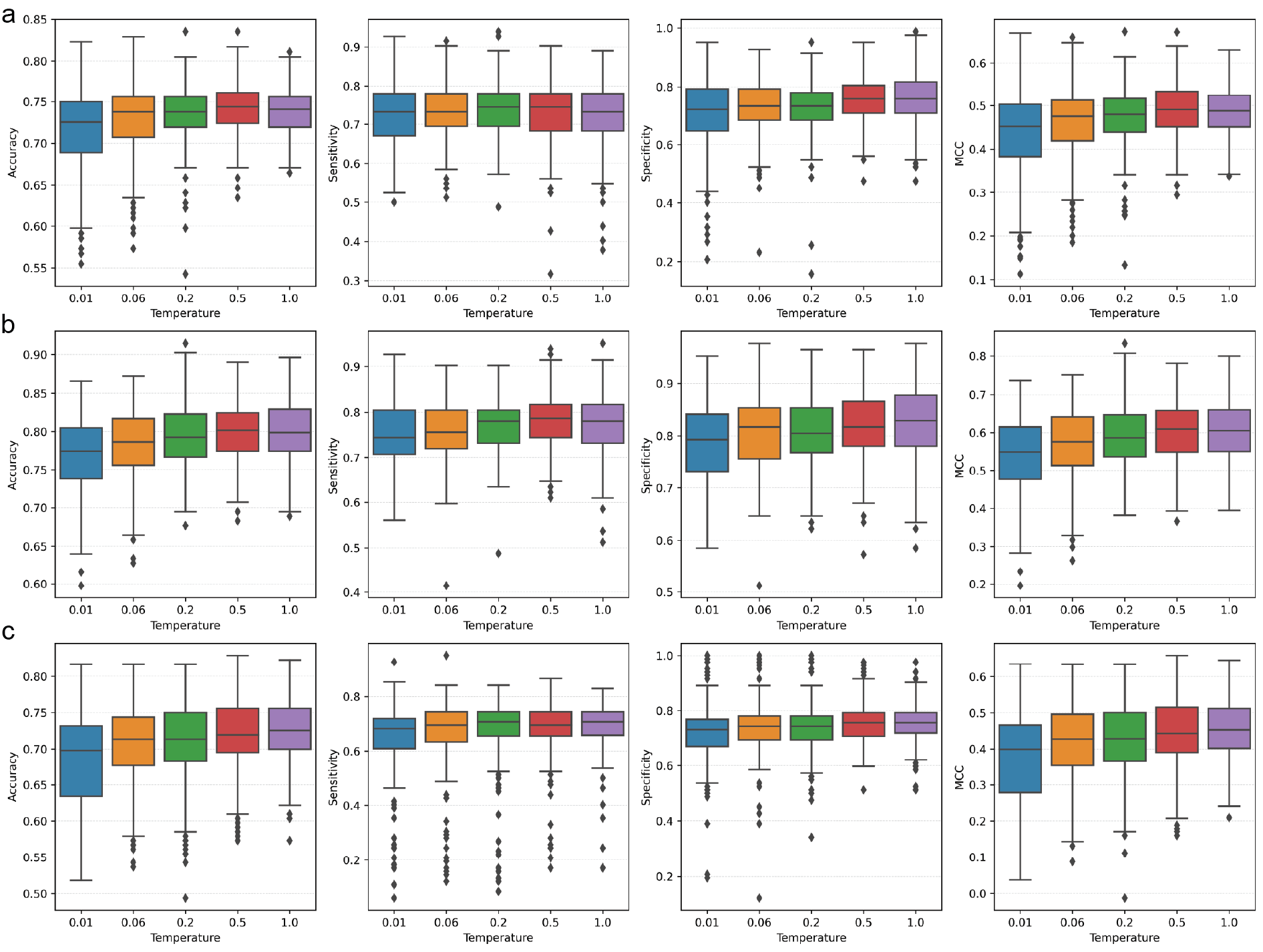


**Figure S3. Performance comparison relative to temperature coefficients using the ACP500+ACP164 dataset.** Each bar plot denotes the mean of each performance metric trained by contrastive learning relative to the coefficient temperature. Error bars represent ± standard error of the mean. The results are displayed by encoder architectures of **(a)** convolutional neural network (CNN), **(b)** transformer-encoder, and **(c)** long short-term memory (LSTM).


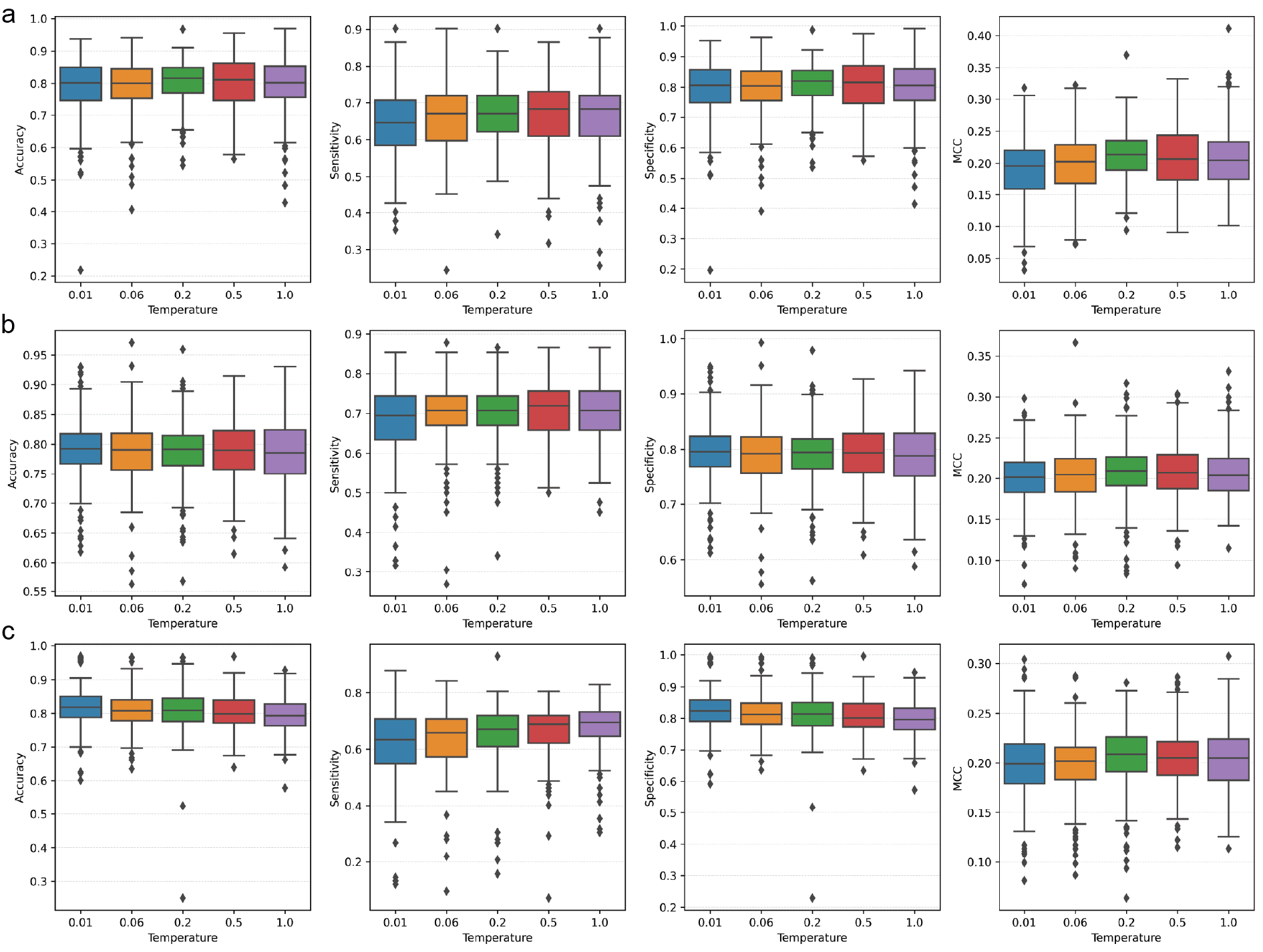


**Figure S4. Performance comparison relative to temperature coefficients using the ACP500+ACP2710 dataset.** Each bar plot denotes the mean of each performance metric trained by contrastive learning relative to the coefficient temperature. Error bars represent ± standard error of the mean. The results are displayed by encoder architectures of **(a)** convolutional neural network (CNN), **(b)** transformer-encoder, and **(c)** long short-term memory (LSTM).

**
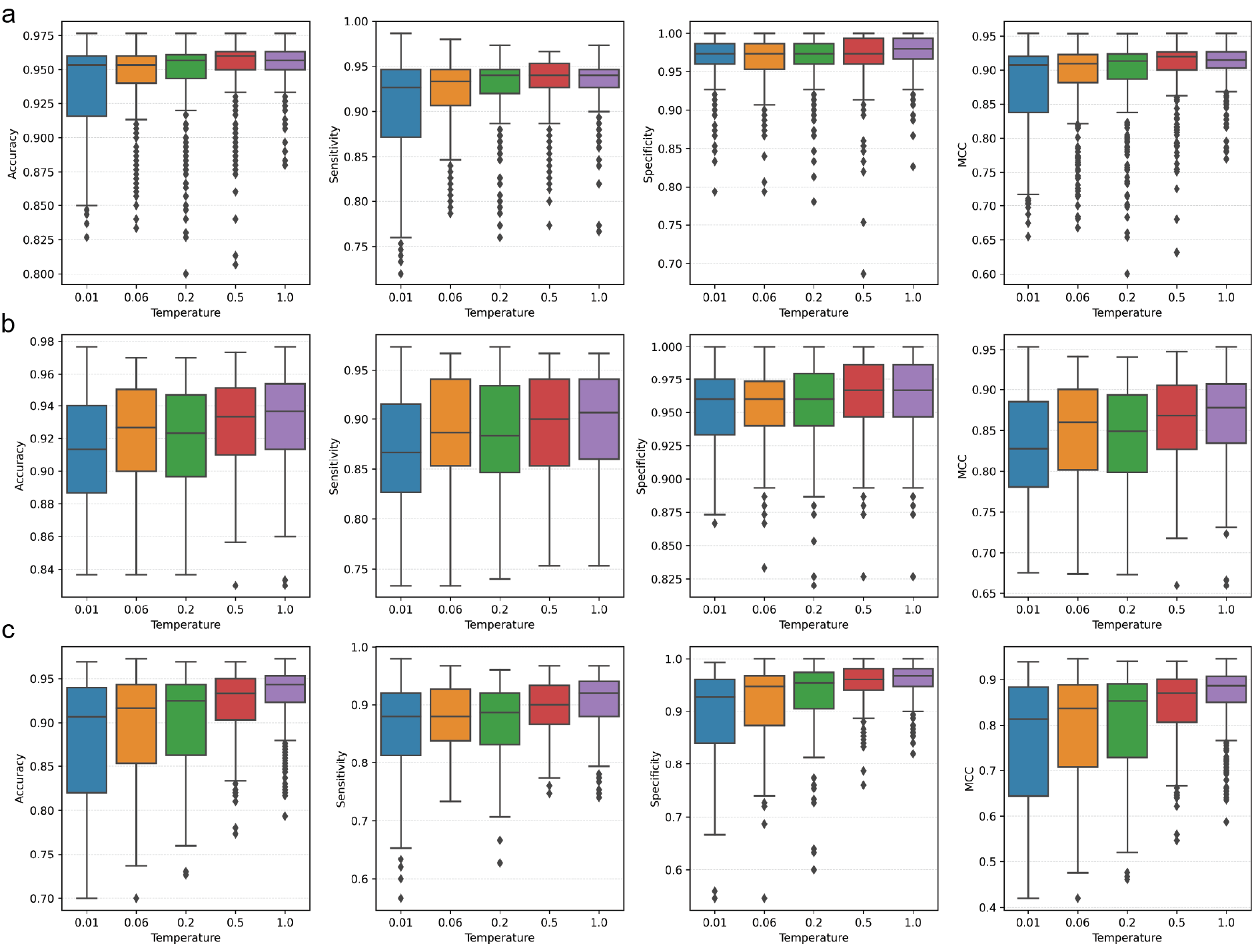
**

**Figure S5. Performance comparison relative to temperature coefficients using the LEE+Independent dataset.** Each bar plot denotes the mean of each performance metric trained by contrastive learning relative to the coefficient temperature. Error bars represent ± standard error of the mean. The results are displayed by encoder architectures of **(a)** convolutional neural network (CNN), **(b)** transformer-encoder, and **(c)** long short-term memory (LSTM).


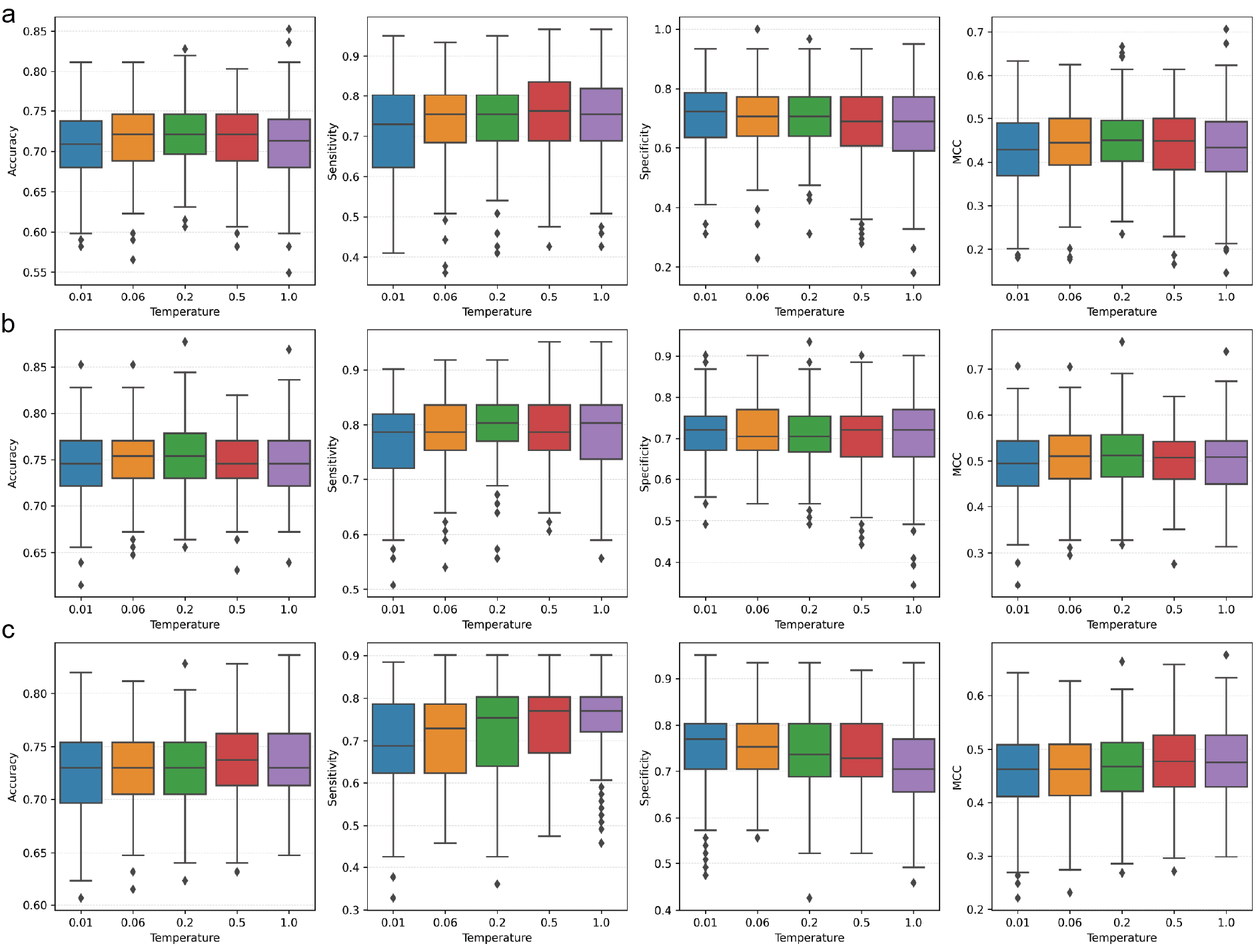


**Figure S6. Performance comparison relative to temperature coefficients using the ACP-Mixed-80 dataset.** Each bar plot denotes the mean of each performance metric trained by contrastive learning relative to the coefficient temperature. Error bars represent ± standard error of the mean. The results are displayed by encoder architectures of **(a)** convolutional neural network (CNN), **(b)** transformer-encoder, and **(c)** long short-term memory (LSTM).


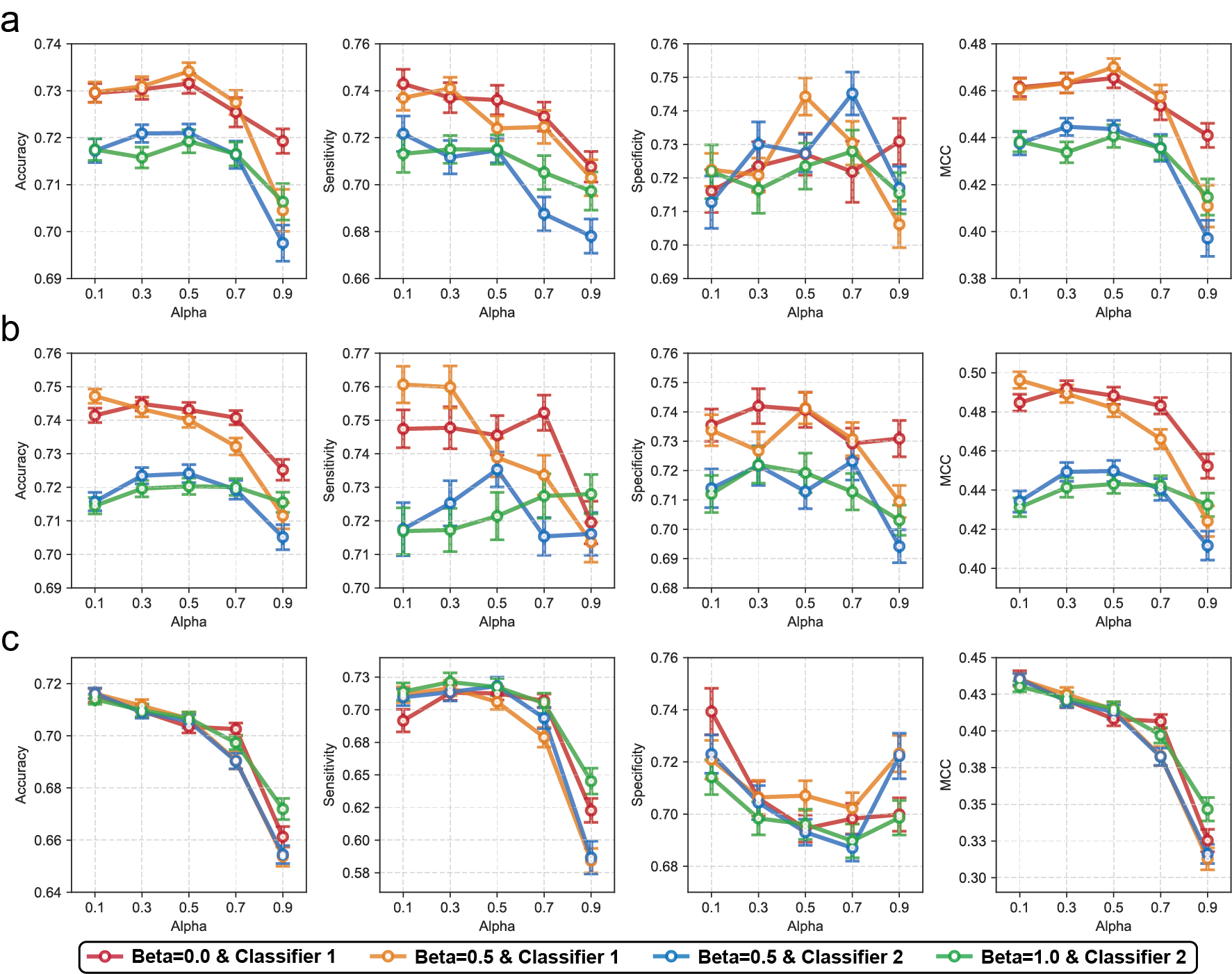


**Figure S7. Performance comparison relative to contrastive learning hyperparameters using the ACP2.0 main dataset.** Each point denotes the mean of each performance metric trained by contrastive learning relative to the coefficient alpha. The results are plotted on each line corresponding to coefficient beta and classifier type. Error bars represent ± standard error of the mean. The results are displayed by encoder architectures of **(a)** convolutional neural network (CNN), **(b)** transformer-encoder, and **(c)** long short-term memory (LSTM).


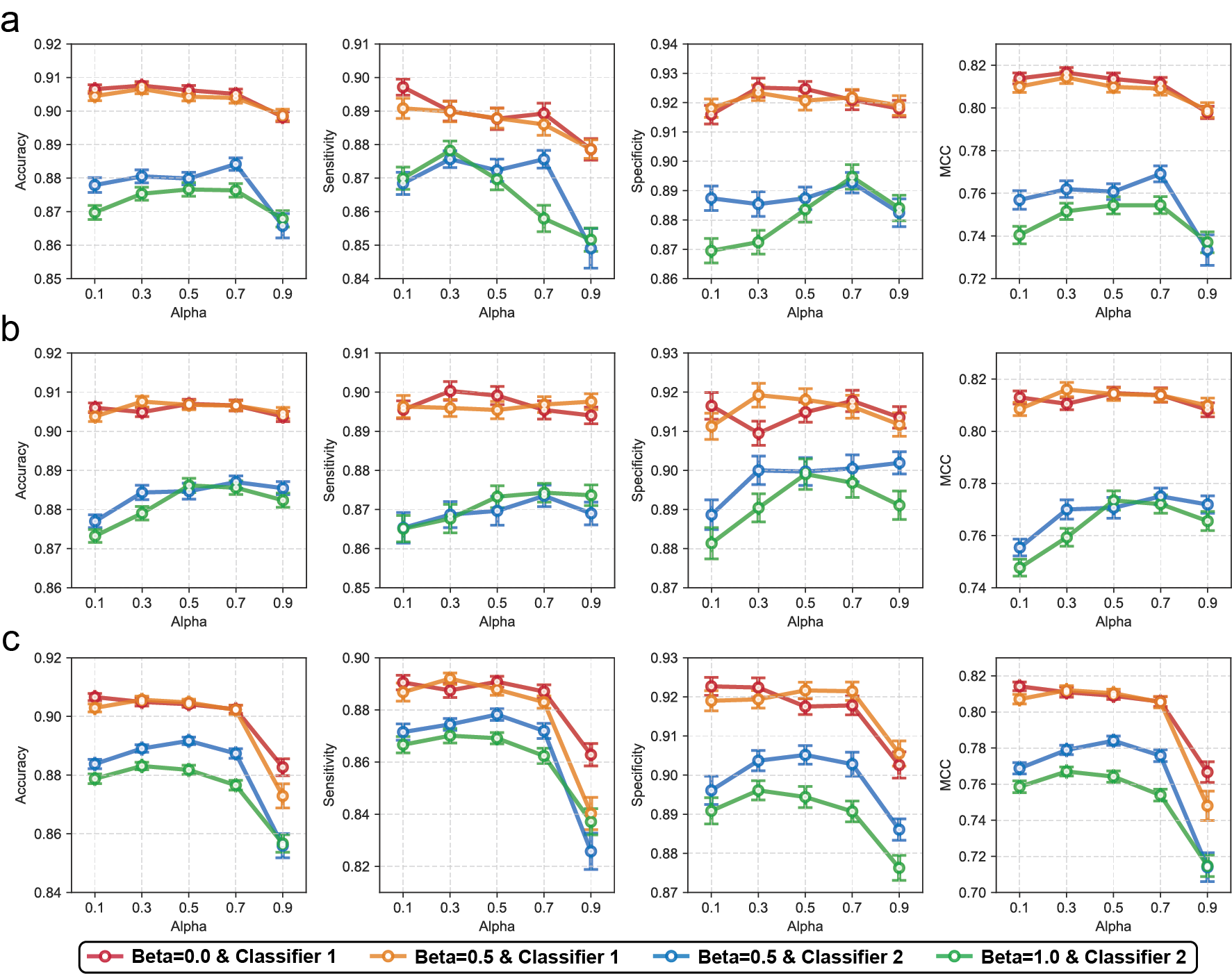


**Figure S8. Performance comparison relative to contrastive learning hyperparameters using the ACP2.0 alternative dataset.** Each point denotes the mean of each performance metric trained by contrastive learning relative to the coefficient alpha. The results are plotted on each line corresponding to coefficient beta and classifier type. Error bars represent ± standard error of the mean. The results are displayed by encoder architectures of **(a)** CNN, **(b)** transformer-encoder, and **(c)** LSTM.


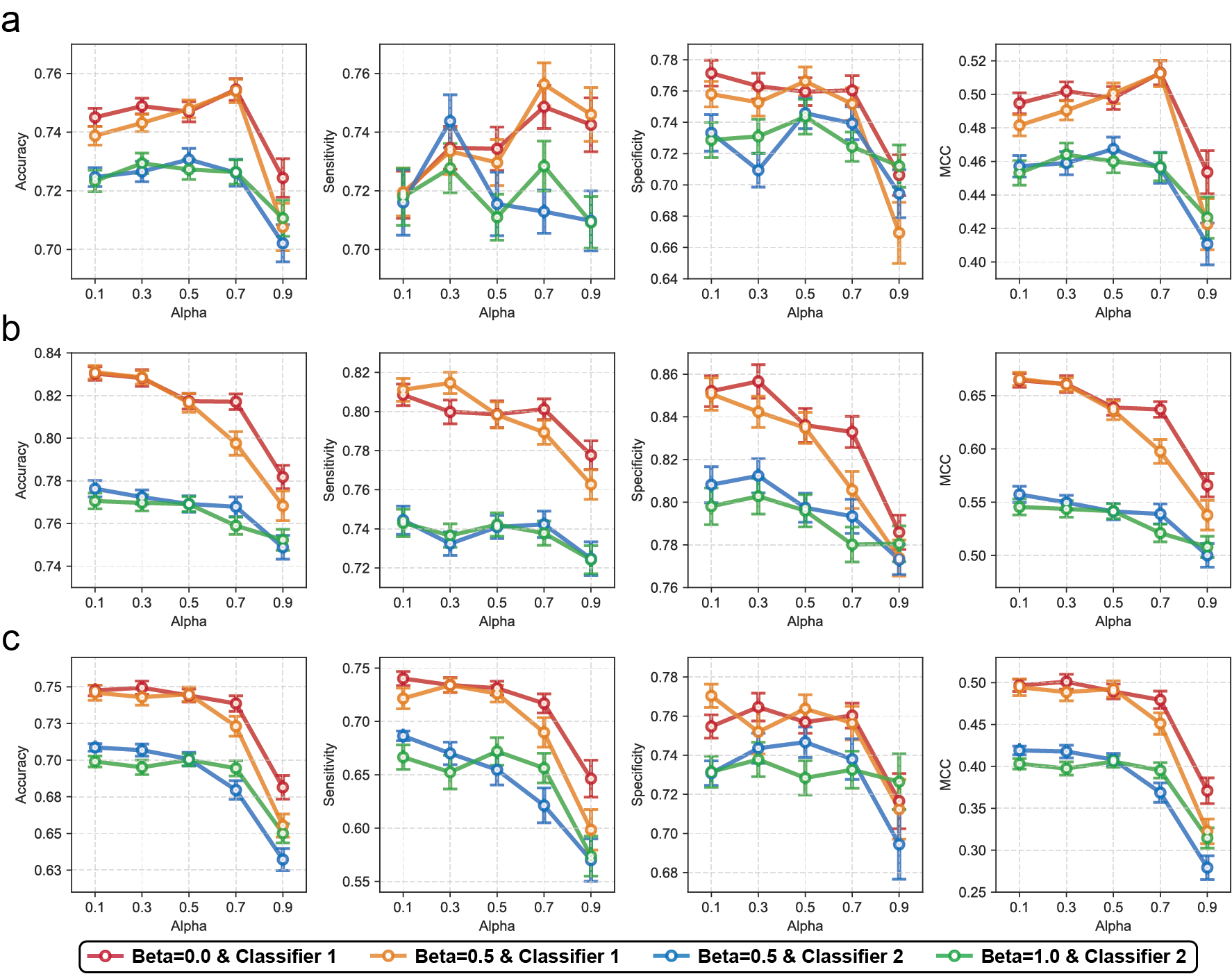


**Figure S9. Performance comparison relative to contrastive learning hyperparameters using the ACP500+ACP164 dataset.** Each point denotes the mean of each performance metric trained by contrastive learning relative to the coefficient alpha. The results are plotted on each line corresponding to coefficient beta and classifier type. Error bars represent ± standard error of the mean. The results are displayed by encoder architectures of **(a)** CNN, **(b)** transformer-encoder, and **(c)** LSTM.

**
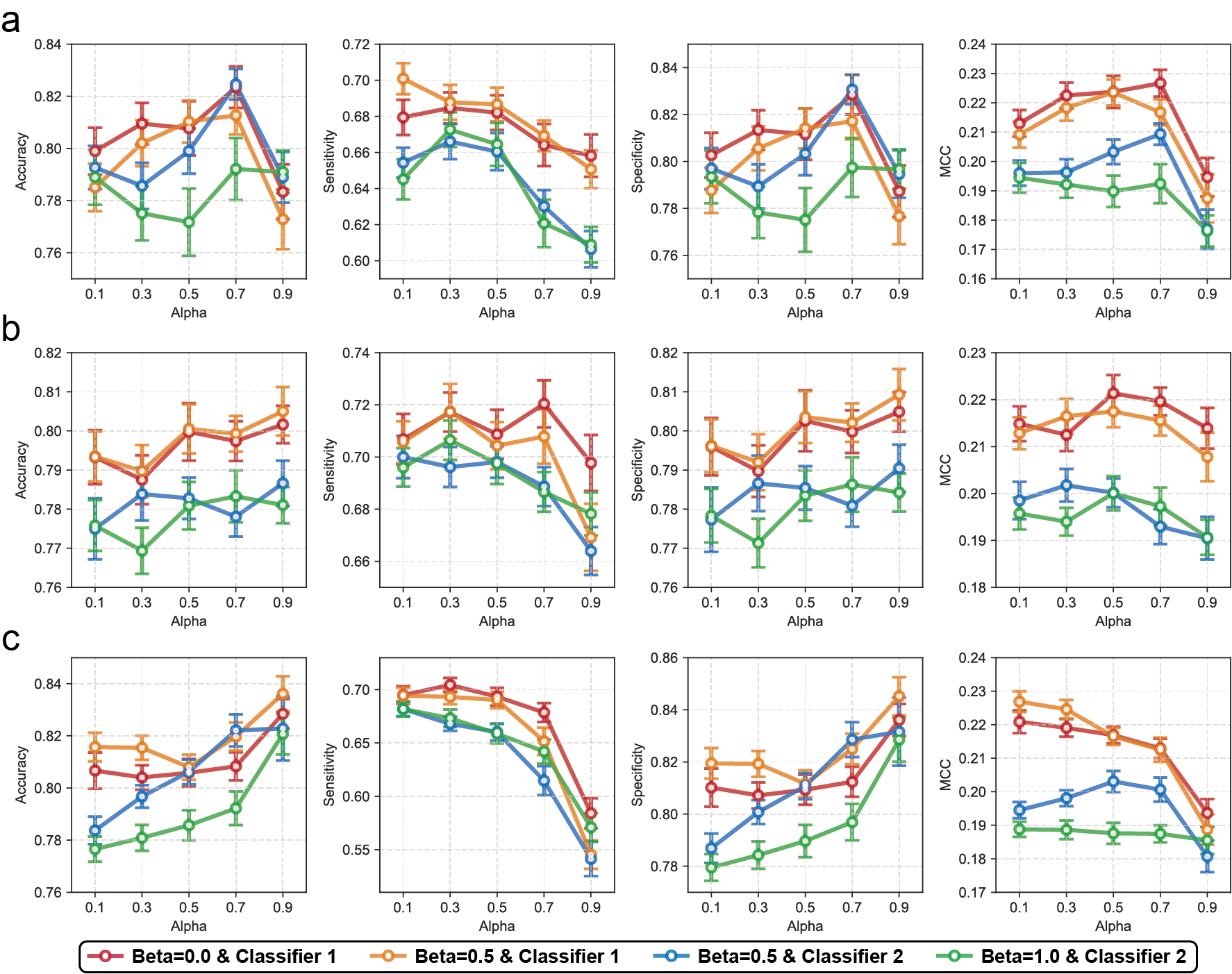
**

**Figure S10. Performance comparison relative to contrastive learning hyperparameters using the ACP500+ACP2710 dataset.** Each point denotes the mean of each performance metric trained by contrastive learning relative to the coefficient alpha. The results are plotted on each line corresponding to coefficient beta and classifier type. Error bars represent ± standard error of the mean. The results are displayed by encoder architectures of **(a)** CNN, **(b)** transformer-encoder, and **(c)** LSTM.


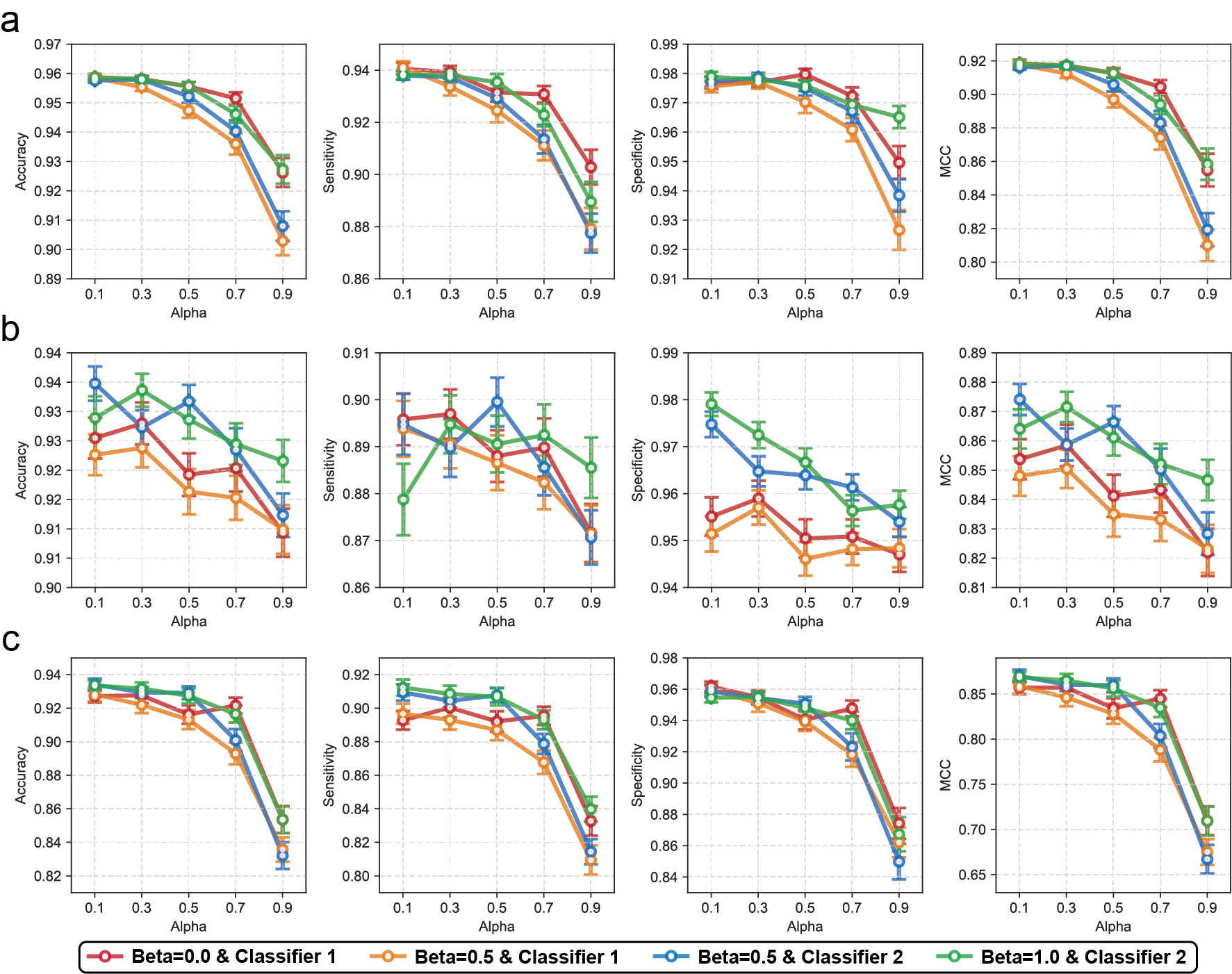


**Figure S11. Performance comparison relative to contrastive learning hyperparameters using the LEE+Independent dataset.** Each point denotes the mean of each performance metric trained by contrastive learning relative to the coefficient alpha. The results are plotted on each line corresponding to coefficient beta and classifier type. Error bars represent ± standard error of the mean. The results are displayed by encoder architectures of **(a)** CNN, **(b)** transformer-encoder, and **(c)** LSTM.


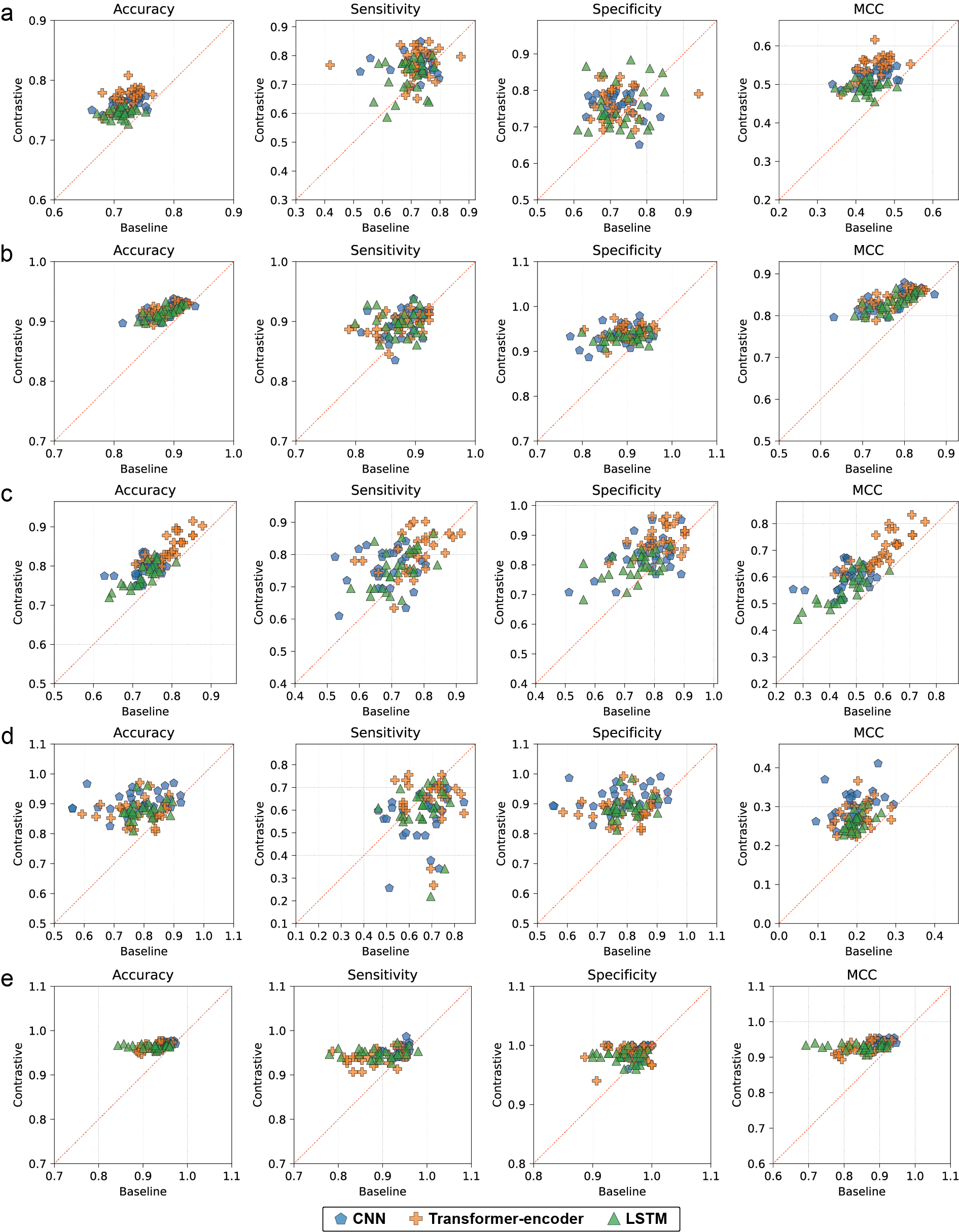


**Figure S12. Performance comparison across training methods with identical architectures and initial weights.** Each scatter represents model performance trained by cross-entropy (baseline) loss and contrastive learning, displaying performance on the respective x- and y-axis. The red dotted line indicates equivalent performance between baseline and contrastive learning. For the identical model architecture and initial model weight, the area above the line signifies superior performance by contrastive learning, and the area below suggests better performance by the baseline. The results were displayed using the (a) AntiCP2.0 main, (b) AntiCP2.0 alternative, (c) ACP500+ACP164, (d) ACP500+ACP2710, and (e) LEE+Independent dataset.


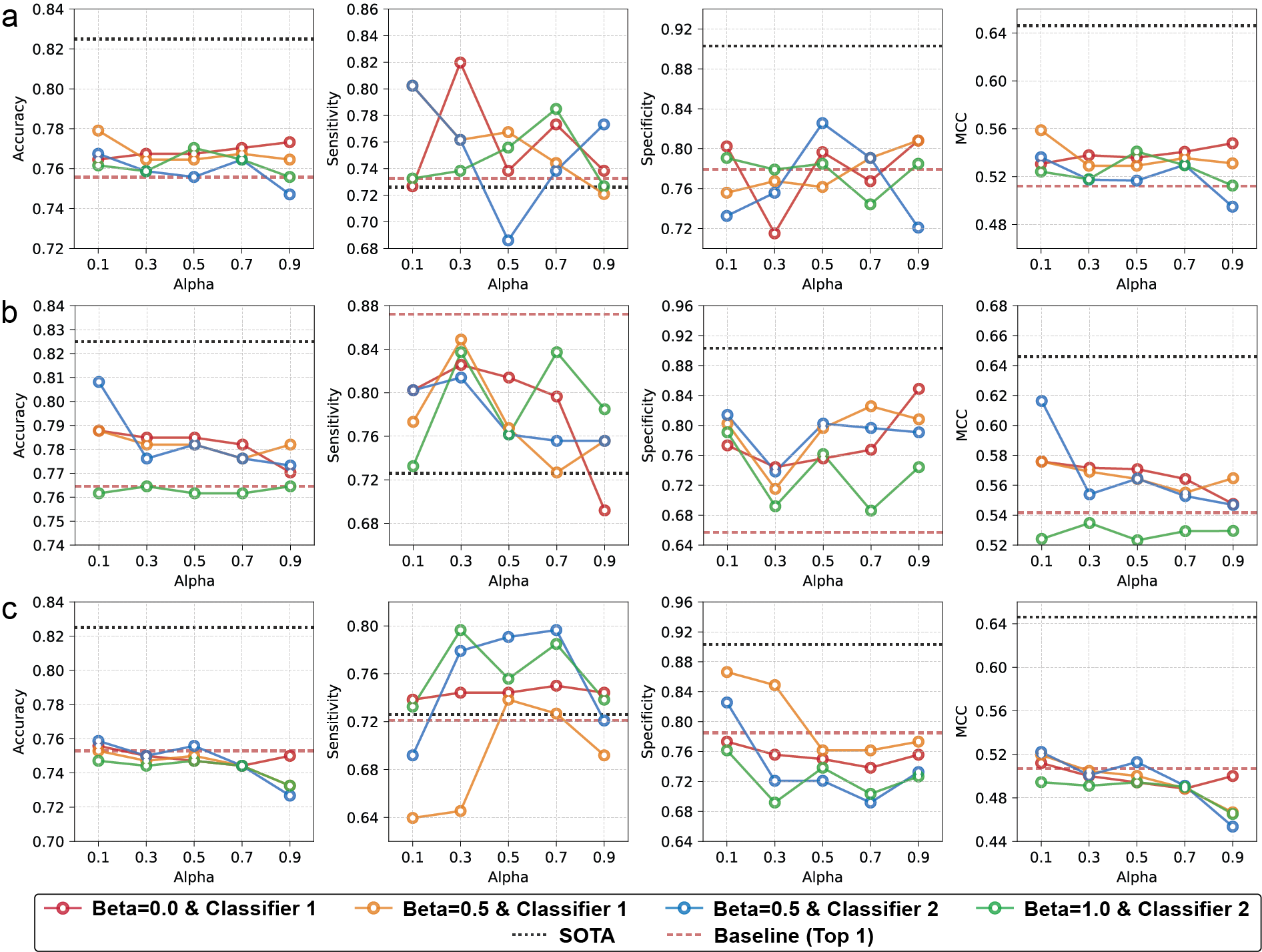


**Figure S13. The best model performance relative to each coefficient alpha and beta using the AntiCP2.0 main dataset.** The best model was selected based on the alpha and beta coefficients that achieved the highest MCC metric values. Line plots are presented for each beta coefficient and prediction classifier per coefficient alpha. Dashed lines represent the SOTA and best performances of the baseline. The results are displayed based on the encoder architectures of **(a)** CNN, **(b)** transformer-encoder, and **(c)** LSTM.


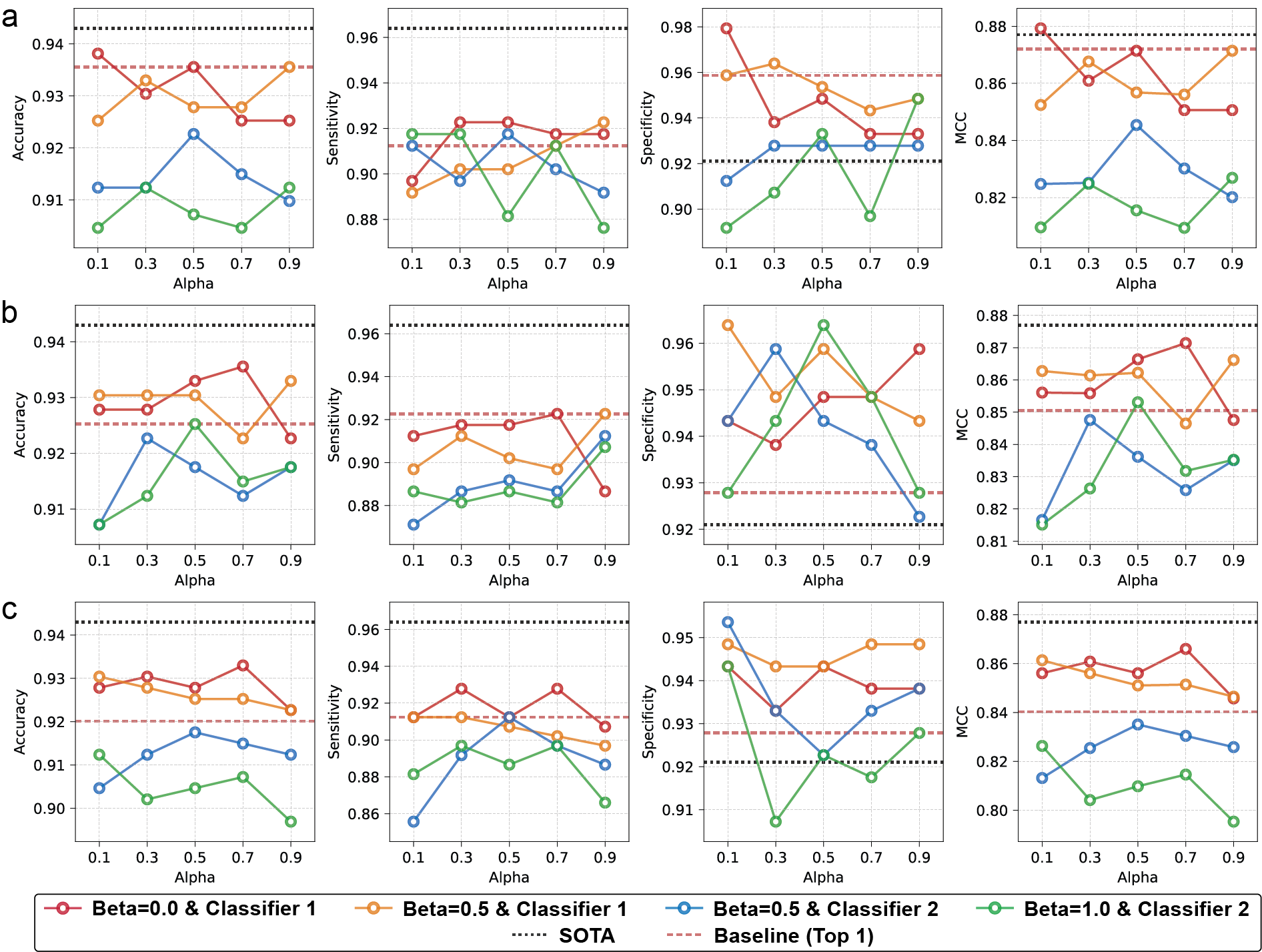


**Figure S14. The best model performance relative to each coefficient alpha and beta using the AntiCP2.0 alternative dataset.** The best model was selected based on the alpha and beta coefficients that achieved the highest MCC metric values. Line plots are presented for each beta coefficient and prediction classifier per coefficient alpha. Dashed lines represent the SOTA and best performances of the baseline. The results are displayed based on the encoder architectures of **(a)** CNN, **(b)** transformer-encoder, and **(c)** LSTM.


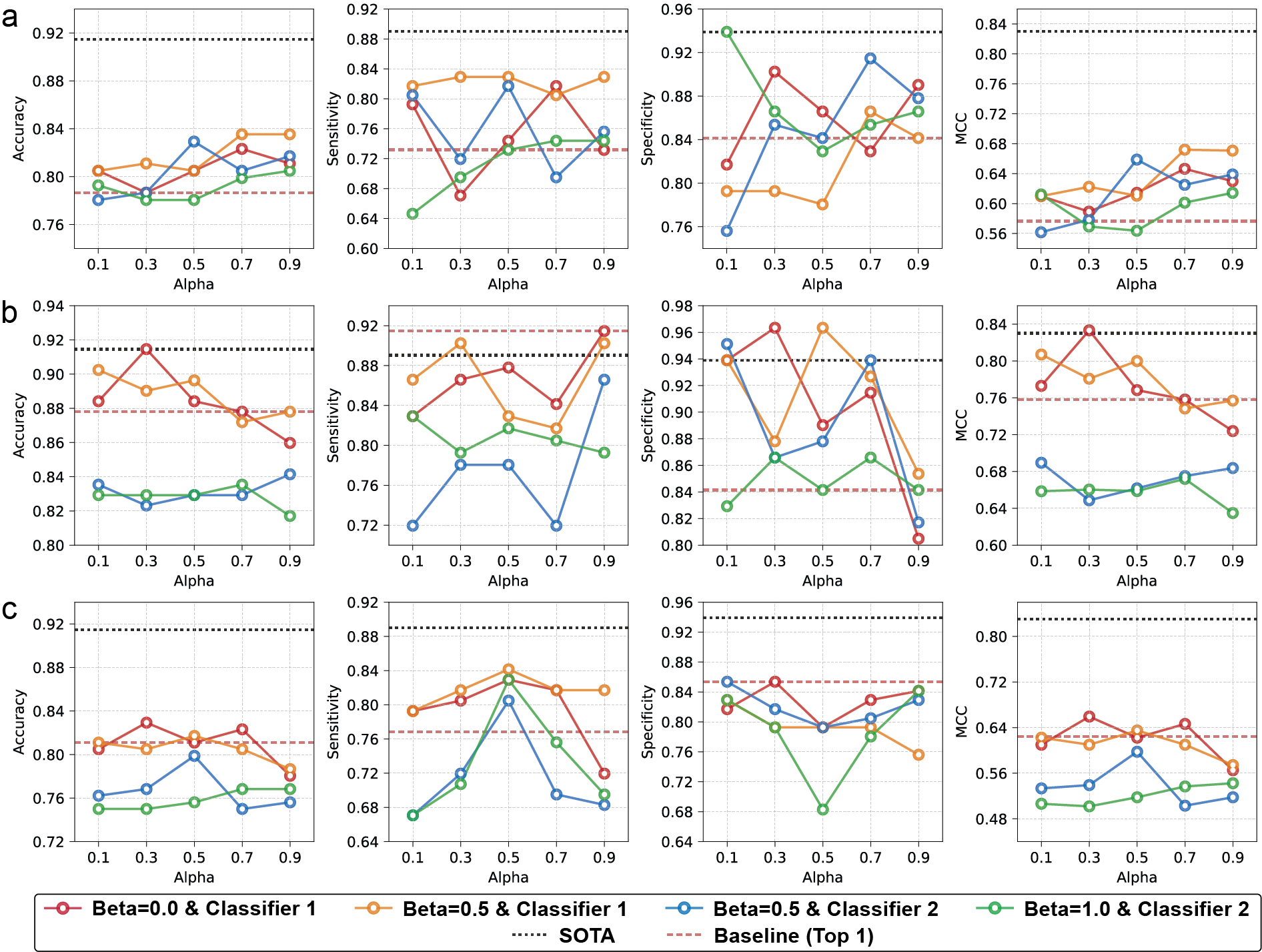


**Figure S15. The best model performance relative to each coefficient alpha and beta using the ACP500+ACP164 dataset.** The best model was selected based on the alpha and beta coefficients that achieved the highest MCC metric values. Line plots are presented for each beta coefficient and prediction classifier per coefficient alpha. Dashed lines represent the SOTA and best performances of the baseline. The results are displayed based on the encoder architectures of **(a)** CNN, **(b)** transformer-encoder, and **(c)** LSTM.


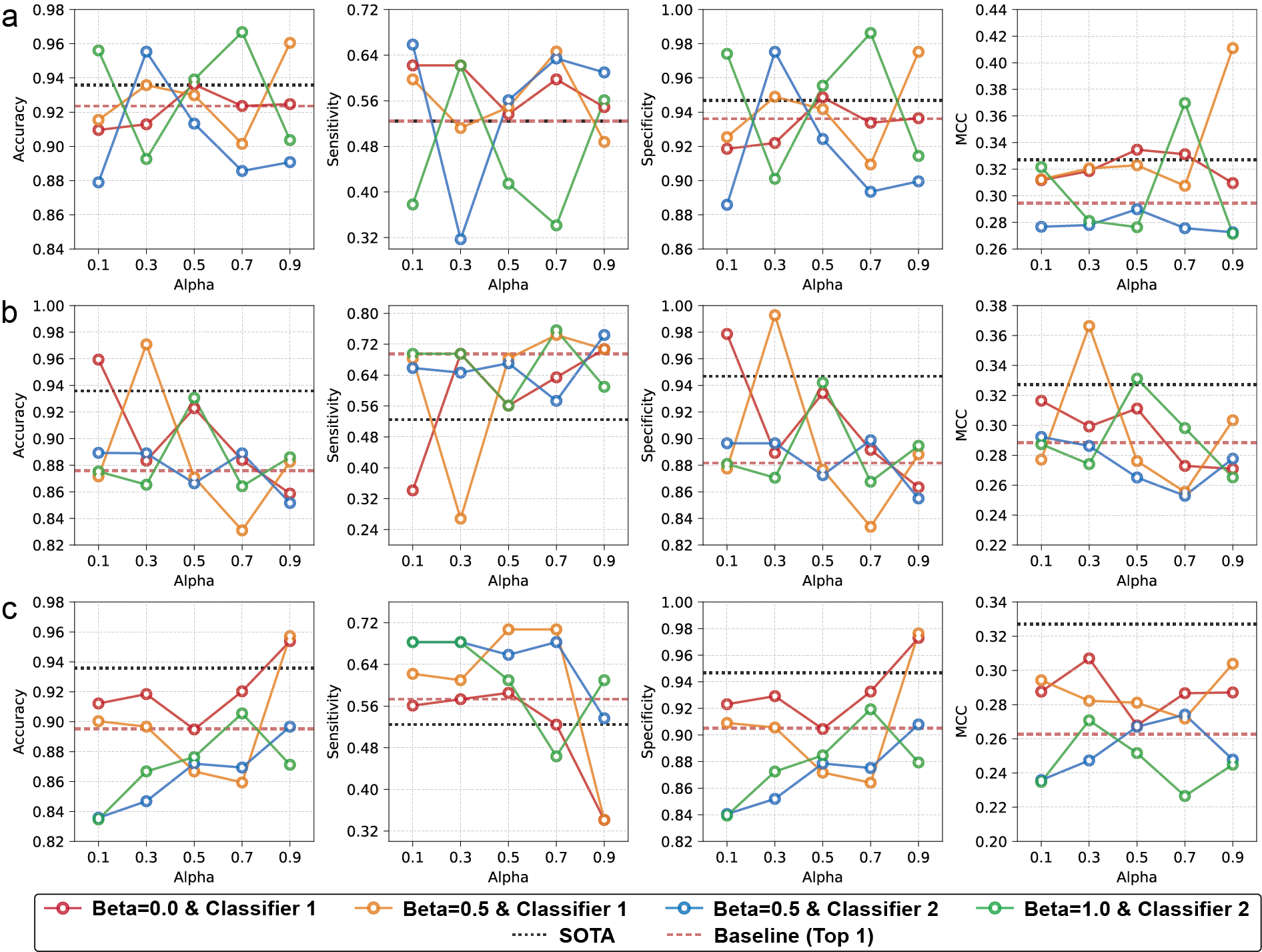


**Figure S16. The best model performance relative to each coefficient alpha and beta using the ACP500+ACP2710 dataset.** The best model was selected based on the alpha and beta coefficients that achieved the highest MCC metric values. Line plots are presented for each beta coefficient and prediction classifier per coefficient alpha. Dashed lines represent the SOTA and best performances of the baseline. The results are displayed based on the encoder architectures of **(a)** CNN, **(b)** transformer-encoder, and **(c)** LSTM.


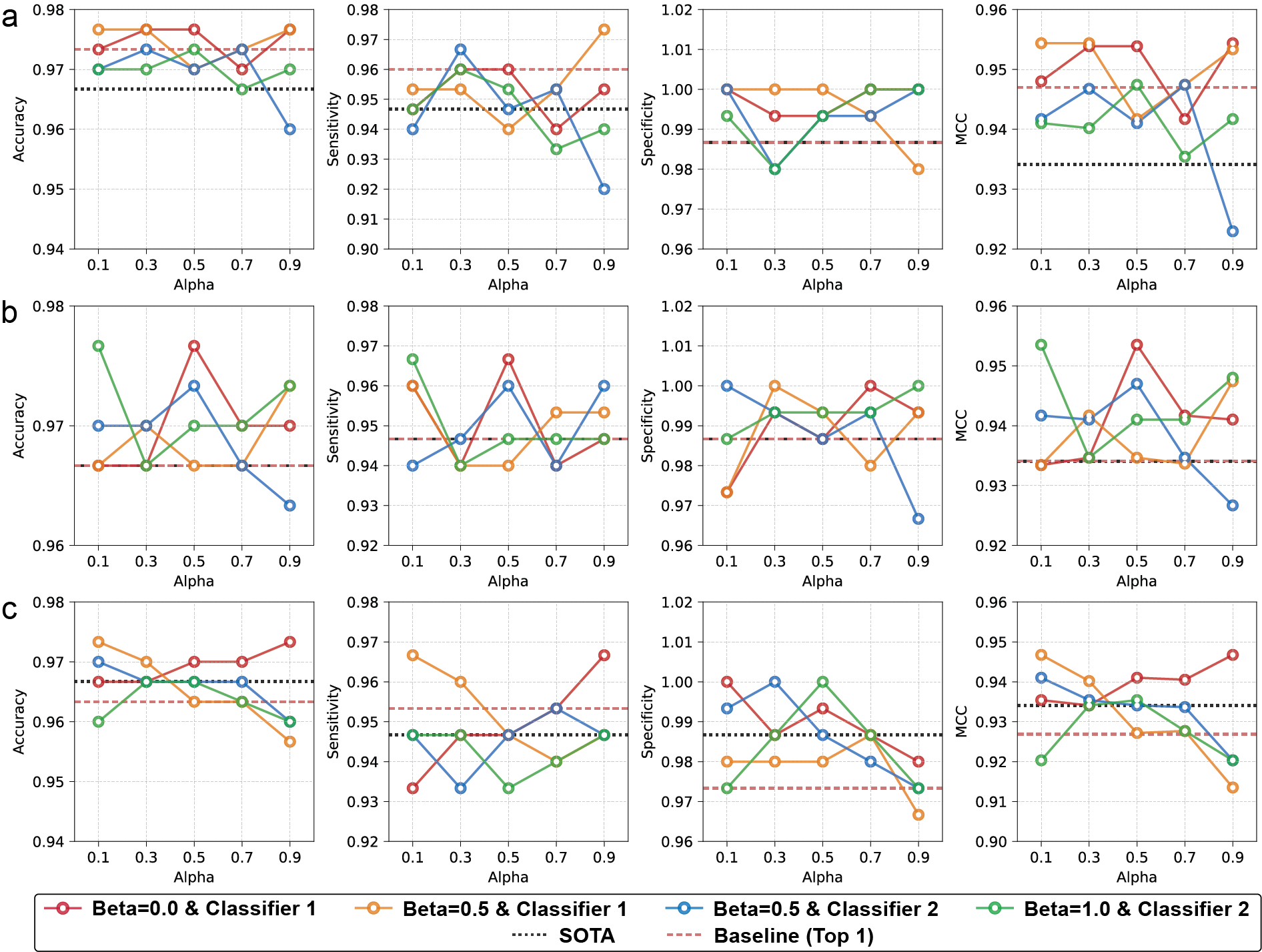


**Figure S17. The best model performance relative to each coefficient alpha and beta using the LEE+Independent dataset.** The best model was selected based on the alpha and beta coefficients that achieved the highest MCC metric values. Line plots are presented for each beta coefficient and prediction classifier per coefficient alpha. Dashed lines represent the SOTA and best performances of the baseline. The results are displayed based on the encoder architectures of **(a)** CNN, **(b)** transformer-encoder, and **(c)** LSTM.

**
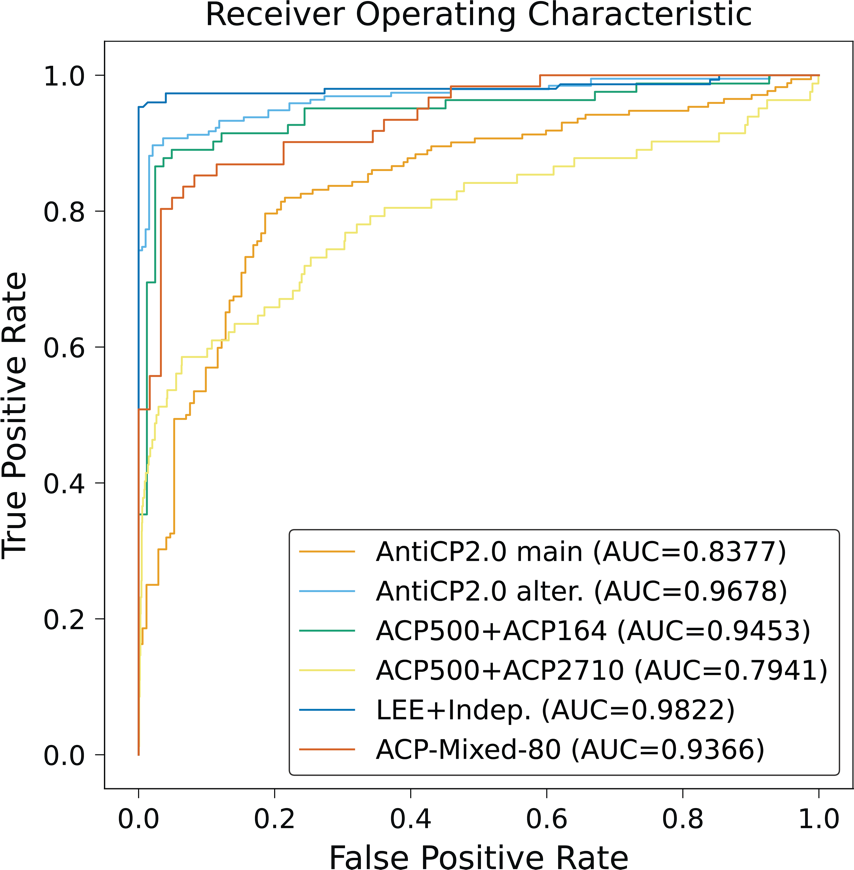
**

**Figure S18. Receiver operating characteristic (ROC) curves of optimal models on benchmark datasets.** The ROC curve was plotted using test data from the corresponding benchmark datasets, with the area under the curve (AUC) indicated in the legend.

**
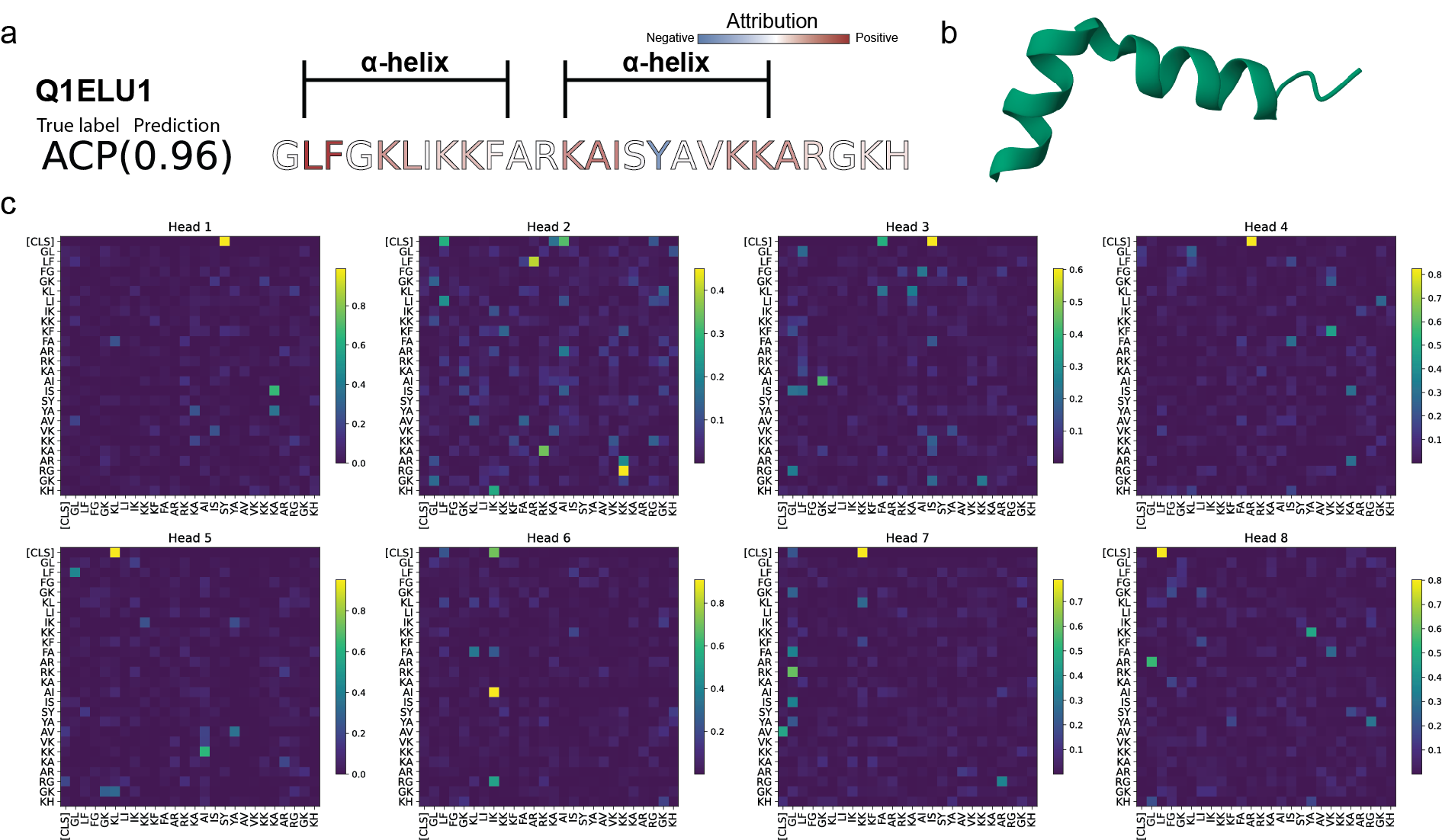
**

**Figure S19. Model interpretability study using the ACP-Mixed-80 test dataset.** (a) An example of attribution for M-zodatoxin-Lt2a (Uniprot ID: Q1ELU1) is provided. The model predicted the peptide as an ACP with a 0.96 probability. (b) The peptide structure of M-zodatoxin-Lt2a obtained from the PDB database (PDB ID: 2G9P) is depicted. (c) Attention scores from various attention heads of the first transformer-encoder layer are presented.

**Table S1. Number of peptides for model train and test**

| Dataset | Train negative | Train positive | Test negative | Test positive | Total |
| --- | --- | --- | --- | --- | --- |
| ACP2.0 main | 689 | 689 | 172 | 172 | 1,722 |
| ACP2.0 alternative | 776 | 776 | 194 | 194 | 1,940 |
| LEE + Independent | 422 | 422 | 150 | 150 | 1,144 |
| ACP500+ACP2710 | 250 | 250 | 2,628 | 82 | 3,210 |
| ACP500+ACP164 | 250 | 250 | 82 | 82 | 664 |
| ACP-Mixed-80 | 242 | 242 | 61 | 61 | 606 |

**Table S2. Performance improvement ratios by contrastive learning compared with baseline within identical model architectures and weights**

| Dataset | Encoder | Accuracy | Sensitivity | Specificity | MCC |
| --- | --- | --- | --- | --- | --- |
| ACP-Mixed-80 | CNN | 1.0000 | 0.7500 | 0.7143 | 1.0000 |
|  | Transformer-encoder | 0.9643 | 0.6071 | 0.8571 | 1.0000 |
|  | LSTM | 0.9643 | 0.5714 | 0.7500 | 0.9643 |
| AntiCP2.0 main | CNN | 0.9643 | 0.6429 | 0.8214 | 0.9643 |
|  | Transformer-encoder | 1.0000 | 0.7143 | 0.7857 | 1.0000 |
|  | LSTM | 0.9643 | 0.7500 | 0.6429 | 0.9643 |
| AntiCP2.0 alter. | CNN | 0.9643 | 0.6786 | 0.8571 | 0.9643 |
|  | Transformer-encoder | 1.0000 | 0.6429 | 0.8929 | 1.0000 |
|  | LSTM | 1.0000 | 0.7500 | 0.8571 | 1.0000 |
| ACP500+ACP164 | CNN | 1.0000 | 0.7857 | 0.7143 | 1.0000 |
|  | Transformer-encoder | 1.0000 | 0.5714 | 0.8214 | 1.0000 |
|  | LSTM | 0.9643 | 0.7500 | 0.7857 | 1.0000 |
| ACP500+ACP2710 | CNN | 0.9286 | 0.2500 | 0.9286 | 1.0000 |
|  | Transformer-encoder | 0.8929 | 0.3214 | 0.8929 | 0.9643 |
|  | LSTM | 0.9643 | 0.3214 | 0.9643 | 1.0000 |
| LEE+Independent | CNN | 0.9643 | 0.7500 | 0.7143 | 0.9643 |
|  | Transformer-encoder | 1.0000 | 0.8214 | 0.7500 | 1.0000 |
|  | LSTM | 1.0000 | 0.8929 | 0.7143 | 1.0000 |

**Table S3. Optimized model performance results for respective model architecture, training strategies, and pre-SOTA**

| Dataset | Model | Alpha | Beta | AA token  length | Accuracy | Sensitivity | Specificity | MCC | Precision |
| --- | --- | --- | --- | --- | --- | --- | --- | --- | --- |
| ACP-Mixed-80 | ACP-MLC [1] |  |  |  | 0.7868 | 0.8032 | 0.7705 | 0.5741 | 0.7780 |
|  | ACPred-LAF [2] | - | - | - | 0.8361 | 0.8033 | 0.8689 | 0.6736 | - |
|  | CNN (ours) | 0 | 0 | 1 | 0.7869 | 0.8197 | 0.7541 | 0.5750 | 0.7692 |
|  |  | 0.9 | 0.5 | 1 | 0.8525 | 0.8197 | 0.8852 | 0.7064 | 0.8772 |
|  | Transformer-encoder (ours) | 0 | 1 | 2 | **0.8770** | 0.8361 | 0.9180 | 0.7566 | 0.9107 |
|  |  | 0.1 | 0.5 | 2 | **0.8770** | 0.8197 | **0.9344** | **0.7591** | **0.9259** |
|  | LSTM (ours) | 0 | 1 | 2 | 0.8115 | 0.8689 | 0.7541 | 0.6271 | 0.7794 |
|  |  | 0.3 | 0.5 | 2 | 0.8361 | **0.8852** | 0.7869 | 0.6754 | 0.8060 |
| AntiCP 2.0 main | ACP-MHCNN [3] | - | - | - | 0.7300 | 0.7850 | 0.6740 | 0.4600 | 0.7060 |
|  | ACP-OPE [4] | - | - | - | 0.7895 | 0.8153 | 0.7676 | - | - |
|  | ACPred-FL [5] | - | - | - | 0.4480 | 0.6705 | 0.2254 | -0.1200 | - |
|  | ACPred-LAF [2] | - | - | - | 0.7907 | 0.8198 | 0.7616 | 0.5824 | - |
|  | AntiCP 2.0 [6] | - | - | - | 0.7543 | 0.7746 | 0.7341 | 0.5100 | - |
|  | AntiCP [7] | - | - | - | 0.5058 | **1.0000** | 0.0116 | 0.0700 | - |
|  | iACP-DRLF [8] | - | - | - | 0.7749 | 0.8070 | 0.7427 | 0.5510 | - |
|  | iACP-FSCM [9] | - | - | - | **0.8250** | 0.7260 | **0.9030** | **0.6460** | - |
|  | iACP-MultiCNN [10] | - | - | - | 0.8050 | 0.8230 | 0.7870 | 0.6010 | - |
|  | CNN (ours) | 0 | 0 | 1 | 0.7558 | 0.7326 | 0.7791 | 0.5122 | 0.7683 |
|  |  | 0.1 | 0.5 | 1 | 0.7791 | 0.8023 | 0.7558 | 0.5587 | 0.7667 |
|  | Transformer-encoder (ours) | 0 | 0 | 1 | 0.7645 | 0.8721 | 0.6570 | 0.5418 | 0.7177 |
|  |  | 0.1 | 0.5 | 2 | 0.8081 | 0.8023 | 0.8140 | 0.6163 | **0.8118** |
|  | LSTM (ours) | 0 | 1 | 2 | 0.7529 | 0.7209 | 0.7849 | 0.5069 | 0.7702 |
|  |  | 0.1 | 0.5 | 2 | 0.7587 | 0.6919 | 0.8256 | 0.5221 | 0.7987 |
| AntiCP 2.0 alternative  Dataset | ACP-MHCNN [3] | - | - | - | 0.9000 | 0.8660 | 0.9430 | 0.8100 | 0.9380 |
|  | ACPred-FL [5] | - | - | - | 0.4380 | 0.6021 | 0.2558 | -0.1500 | - |
|  | ACPred-LAF [2] | - | - | - | 0.9021 | 0.8918 | 0.9124 | 0.8043 | - |
|  | AntiCP 2.0 [6] | - | - | - | 0.9201 | 0.9227 | 0.9175 | 0.8400 | - |
|  | AntiCP [7] | - | - | - | 0.8995 | 0.8969 | 0.9020 | 0.8000 | - |
|  | iACP-DRLF [8] | - | - | - | 0.9300 | 0.8960 | 0.9640 | 0.8620 | - |
|  | iACP-MultiCNN [10] | - | - | - | **0.9430** | **0.9640** | 0.9210 | 0.8770 | - |
|  | CNN (ours) | 0 | 0 | 1 | 0.9356 | 0.9124 | 0.9588 | 0.8721 | 0.9568 |
|  |  | 0.1 | 0 | 1 | 0.9381 | 0.8969 | **0.9794** | **0.8793** | **0.9775** |
|  | Transformer-encoder (ours) | 0 | 0 | 1 | 0.9253 | 0.9227 | 0.9278 | 0.8505 | 0.9275 |
|  |  | 0.7 | 0 | 1 | 0.9356 | 0.9227 | 0.9485 | 0.8714 | 0.9471 |
|  | LSTM (ours) | 0 | 0 | 1 | 0.9201 | 0.9124 | 0.9278 | 0.8403 | 0.9267 |
|  |  | 0.7 | 0 | 1 | 0.9330 | 0.9278 | 0.9381 | 0.8660 | 0.9375 |
| ACP500 + ACP164 | ACP-DL [11] | - | - | - | 0.8470 | 0.8900 | 0.8050 | 0.6200 | - |
|  | ACP-MHCNN [3] | - | - | - | 0.9100 | **0.9760** | 0.8420 | 0.8200 | 0.8600 |
|  | ACPred-FL [5] | - | - | - | 0.9020 | 0.8290 | **0.9760** | 0.8140 | - |
|  | ACPred-LAF [2] | - | - | - | **0.9146** | 0.8902 | 0.9390 | 0.8303 | - |
|  | CNN (ours) | 0 | 0 | 1 | 0.7866 | 0.7317 | 0.8415 | 0.5767 | 0.8219 |
|  |  | 0.7 | 0.5 | 1 | 0.8354 | 0.8049 | 0.8659 | 0.6720 | 0.8571 |
|  | Transformer-encoder (ours) | 0 | 0 | 1 | 0.8780 | 0.9146 | 0.8415 | 0.7581 | 0.8523 |
|  |  | 0.3 | 0 | 1 | **0.9146** | 0.8659 | 0.9634 | **0.8332** | **0.9595** |
|  | LSTM (ours) | 0 | 0 | 1 | 0.8110 | 0.7683 | 0.8537 | 0.6242 | 0.8400 |
|  |  | 0.3 | 0 | 1 | 0.8293 | 0.8049 | 0.8537 | 0.6593 | 0.8462 |
| ACP500 + ACP2710 | ACPred-FL [5] | - | - | - | 0.8530 | 0.6950 | 0.8580 | 0.2590 | - |
|  | ACPred-Fuse [12] | - | - | - | 0.8900 | **0.7200** | 0.8950 | 0.3200 | - |
|  | ACPred-LAF [2] | - | - | - | 0.9358 | 0.5244 | 0.9468 | 0.3271 | - |
|  | iACP [13] | - | - | - | 0.8770 | 0.5490 | 0.8880 | 0.2260 | - |
|  | PEPred-Suite [14] | - | - | - | 0.8990 | 0.6830 | 0.9060 | 0.3200 | - |
|  | CNN (ours) | 0 | 0 | 1 | 0.9236 | 0.5244 | 0.9361 | 0.2944 | 0.2038 |
|  |  | 0.9 | 0.5 | 1 | 0.9605 | 0.4878 | 0.9753 | **0.4110** | 0.3810 |
|  | Transformer-encoder (ours) | 0 | 0 | 1 | 0.8760 | 0.6951 | 0.8817 | 0.2884 | 0.1549 |
|  |  | 0.3 | 0.5 | 1 | **0.9708** | 0.2683 | **0.9928** | 0.3664 | **0.5366** |
|  | LSTM (ours) | 0 | 0 | 1 | 0.8952 | 0.5732 | 0.9053 | 0.2627 | 0.1588 |
|  |  | 0.3 | 0 | 1 | 0.9185 | 0.5732 | 0.9292 | 0.3070 | 0.2017 |
| LEE + Independent | ACPred-LAF [2] | - | - | - | 0.9667 | 0.9467 | 0.9867 | 0.9341 | - |
|  | cACP-2LFS [15] | - | - | - | 0.9415 | 0.9133 | 0.9623 | 0.8700 | - |
|  | CNN (ours) | 0 | 1 | 2 | 0.9733 | 0.9600 | 0.9867 | 0.9470 | 0.9863 |
|  |  | 0.1 | 0.5 | 1 | **0.9767** | 0.9533 | **1.0000** | **0.9544** | **1.0000** |
|  | Transformer-encoder (ours) | 0 | 1 | 2 | 0.9667 | 0.9467 | 0.9867 | 0.9341 | 0.9861 |
|  |  | 0.5 | 0 | 1 | **0.9767** | 0.9667 | 0.9867 | 0.9535 | 0.9864 |
|  | LSTM (ours) | 0 | 0 | 1 | 0.9633 | 0.9533 | 0.9733 | 0.9269 | 0.9728 |
|  |  | 0.1 | 0.5 | 1 | 0.9733 | 0.9667 | 0.9800 | 0.9468 | 0.9797 |

*The best and second-best performances were **bolded** and underlined, respectively.

**Table S4. Detailed information of model hyperparameters**

| Encoder type | Embedding dimension | Detailed |
| --- | --- | --- |
| CNN1 | 64 | CNN1D channels: 32, 64, 128 |
| CNN2 | 128 | CNN1D channels: 32, 64, 128 |
| Transformer-encoder 1 | 64 | $d_{ff}$: 256  h: 8  Number of encoder layer: 4 |
| Transformer-encoder 2 | 128 | $d_{ff}$: 512  h: 8  Number of encoder layer: 4 |
| LSTM1 | 64 | Number of hidden nodes: 32  Number of LSTM layers: 3 |
| LSTM2 | 128 | Number of hidden nodes: 32  Number of LSTM layers: 3 |

**Table S5. Detailed information of training hyperparameters**

| Types of training parameters | Parameters |
| --- | --- |
| Dataset | AntiCP2 main, AntiCP2.0 alternative, ACP500+ACP164, ACP500+ACP2710, LEE+Independent, ACP-Mixed-80 |
| Encoder type | CNN1, CNN2, Transformer-encoder1, Transformer-encoder2, LSTM1, LSTM2 |
| Random seed | 7 fixed seed |
| Alpha | 0 (baseline), 0.1, 0.3, 0.5, 0.7, 0.9 |
| Beta | 0, 0.5, 1 |
| Temperature  (Specific to contrastive learning) | 0.01, 0.06, 0.2, 0.5, 1 |
| Learning rate | 1e-3 |

**Supplementary references**

1. Deng H, Ding M, Wang Y et al. ACP-MLC: A two-level prediction engine for identification of anticancer peptides and multi-label classification of their functional types, Computers in Biology and Medicine 2023;158:106844.

2. He W, Wang Y, Cui L et al. Learning embedding features based on multisense-scaled attention architecture to improve the predictive performance of anticancer peptides, Bioinformatics 2021;37:4684-4693.

3. Ahmed S, Muhammod R, Khan ZH et al. ACP-MHCNN: An accurate multi-headed deep-convolutional neural network to predict anticancer peptides, Scientific Reports 2021;11:23676.

4. Yuan Q, Chen K, Yu Y et al. Prediction of anticancer peptides based on an ensemble model of deep learning and machine learning using ordinal positional encoding, Briefings in bioinformatics 2023;24:bbac630.

5. Wei L, Zhou C, Chen H et al. ACPred-FL: a sequence-based predictor using effective feature representation to improve the prediction of anti-cancer peptides, Bioinformatics 2018;34:4007-4016.

6. Agrawal P, Bhagat D, Mahalwal M et al. AntiCP 2.0: an updated model for predicting anticancer peptides, Briefings in bioinformatics 2021;22:bbaa153.

7. Tyagi A, Kapoor P, Kumar R et al. In silico models for designing and discovering novel anticancer peptides, Scientific Reports 2013;3:2984.

8. Lv Z, Cui F, Zou Q et al. Anticancer peptides prediction with deep representation learning features, Briefings in bioinformatics 2021;22:bbab008.

9. Charoenkwan P, Chiangjong W, Lee VS et al. Improved prediction and characterization of anticancer activities of peptides using a novel flexible scoring card method, Scientific Reports 2021;11:3017.

10. Aziz AZB, Hasan MAM, Ahmad S et al. iACP-MultiCNN: Multi-channel CNN based anticancer peptides identification, Analytical Biochemistry 2022;650:114707.

11. Yi H-C, You Z-H, Zhou X et al. ACP-DL: a deep learning long short-term memory model to predict anticancer peptides using high-efficiency feature representation, Molecular Therapy-Nucleic Acids 2019;17:1-9.

12. Rao B, Zhou C, Zhang G et al. ACPred-Fuse: fusing multi-view information improves the prediction of anticancer peptides, Briefings in bioinformatics 2020;21:1846-1855.

13. Chen W, Ding H, Feng P et al. iACP: a sequence-based tool for identifying anticancer peptides, Oncotarget 2016;7:16895.

14. Wei L, Zhou C, Su R et al. PEPred-Suite: improved and robust prediction of therapeutic peptides using adaptive feature representation learning, Bioinformatics 2019;35:4272-4280.

15. Akbar S, Hayat M, Tahir M et al. cACP-2LFS: classification of anticancer peptides using sequential discriminative model of KSAAP and two-level feature selection approach, Ieee Access 2020;8:131939-131948.
